# Supplementary material for: Carm1-arginine methylation of the transcription factor C/EBPα regulates transdifferentiation velocity
Source: eLife. 2023 Jun 27;12:e83951. doi: 10.7554/eLife.83951 (PMC10299824; doi:10.7554/eLife.83951)
Supplement: Figure 3—source data 1. [file elife-83951-fig3-data1.docx]

| **Gene.names** | **UniprotID** | **logFC.p42R35A.over.p42WT** | **p.value.p42R35A.over.p42WT** |
| --- | --- | --- | --- |
| Ncor1 | E9Q2B2 | 2,30 | 0,09 |
| Spi1 | P17433 | 1,80 | 0,02 |
| Ebf1 | Q5SWK4 | 1,02 | 0,28 |
| Elf2 | Q9JHC9 | 0,61 | 0,39 |
| Tbl1xr1 | Q8BHJ5 | 0,53 | 0,41 |
| Tbl1x | Q9QXE7 | 0,48 | 0,39 |
| Fbrs | A0A1N9PTV1 | 0,42 | 0,37 |
| Trim11 | Q99PQ2 | 0,41 | 0,18 |
| Ring1 | O35730 | 0,37 | 0,24 |
| Arid3a | Q62431 | 0,28 | 0,27 |
| Vezf1 | Q5SXC4 | 0,28 | 0,31 |
| Maz | A0A0U1RNL9 | 0,27 | 0,23 |
| Usf1 | Q61069 | 0,26 | 0,13 |
| Wdr5 | P61965 | 0,25 | 0,33 |
| Ets1 | P27577 | 0,23 | 0,14 |
| Actl6a | Q9Z2N8 | 0,22 | 0,61 |
| Stat5a | P42230 | 0,21 | 0,44 |
| Hivep3 | A2A884 | 0,19 | 0,25 |
| Ash2l | Q91X20 | 0,19 | 0,62 |
| Nap1l1 | Q3TF41 | 0,17 | 0,37 |
| Med8 | Q9DAY7 | 0,17 | 0,23 |
| Yaf2 | Q99LW6 | 0,17 | 0,58 |
| Pygo2 | Q80V76 | 0,15 | 0,67 |
| Bcl9 | Q9D219 | 0,15 | 0,42 |
| Ss18 | A0A3Q4EGQ6 | 0,15 | 0,57 |
| Lef1 | Q8BGZ9 | 0,15 | 0,79 |
| Zhx1 | P70121 | 0,14 | 0,45 |
| Arnt | E9QLT6 | 0,14 | 0,13 |
| Gabpb2 | P81069 | 0,14 | 0,52 |
| Jdp2 | P97875 | 0,13 | 0,58 |
| Zfp296 | E9Q6W4 | 0,12 | 0,71 |
| Ep300 | B2RWS6 | 0,12 | 0,42 |
| Tle4;Tle1 | Q62441 | 0,11 | 0,56 |
| Pspc1 | Q8R326 | 0,11 | 0,64 |
| Tle3 | F8WIE2 | 0,10 | 0,59 |
| Nfil3 | O08750 | 0,10 | 0,69 |
| Arid3b | Q9Z1N7 | 0,10 | 0,68 |
| Ipo7 | Q9EPL8 | 0,09 | 0,63 |
| Foxo1 | Q9R1E0 | 0,08 | 0,62 |
| Crebbp | P45481 | 0,08 | 0,24 |
| Jun | P05627 | 0,08 | 0,66 |
| Gmeb1 | Q9JL60 | 0,07 | 0,70 |
| Zmiz1 | Q6P1E1 | 0,07 | 0,68 |
| Lin9 | Q8C735 | 0,07 | 0,62 |
| E2f3 | O35261 | 0,07 | 0,83 |
| Trim33 | E9QP19 | 0,07 | 0,45 |
| Ncoa1 | P70365 | 0,07 | 0,76 |
| Atf7 | Q3TZR9 | 0,07 | 0,48 |
| Mef2a | Q60929 | 0,07 | 0,81 |
| Lef1 | P27782 | 0,07 | 0,71 |
| Pou2f2 | Q00196 | 0,06 | 0,76 |
| Mef2d | Q921S6 | 0,06 | 0,76 |
| Rfwd2 | Q9R1A8 | 0,06 | 0,58 |
| Irf2bp2 | E9Q1P8 | 0,06 | 0,80 |
| Mtf1 | Q07243 | 0,06 | 0,34 |
| Trim24 | Q64127 | 0,06 | 0,27 |
| Hmbox1 | Q8BJA3 | 0,06 | 0,67 |
| Nap1l4 | Q78ZA7 | 0,06 | 0,83 |
| Gabpa | Q00422 | 0,06 | 0,79 |
| Ogt | Q8CGY8 | 0,06 | 0,56 |
| Gopc | Q8BH60 | 0,06 | 0,66 |
| Irf2bpl | Q8K3X4 | 0,05 | 0,87 |
| Rnf2 | Q9CQJ4 | 0,04 | 0,83 |
| Maml3 | D4QGC2 | 0,04 | 0,72 |
| Fam60a | Q8C8M1 | 0,04 | 0,88 |
| L3mbtl3 | Q8BLB7 | 0,04 | 0,81 |
| Brd4 | Q9ESU6 | 0,04 | 0,77 |
| Med13 | Q5SWW4 | 0,04 | 0,71 |
| Cbfa2t3 | O54972 | 0,04 | 0,79 |
| Med4 | Q9CQA5 | 0,04 | 0,83 |
| Hdac1 | O09106 | 0,03 | 0,87 |
| Elf1 | A0A2I3BPX9 | 0,03 | 0,84 |
| Tet2 | Q4JK59 | 0,03 | 0,66 |
| Znf629 | Q6A085 | 0,03 | 0,83 |
| Nfia | B1AUB9 | 0,03 | 0,81 |
| Hhex | P43120 | 0,03 | 0,85 |
| Med15 | E9Q7C1 | 0,03 | 0,67 |
| Ncor2 | F8VQL9 | 0,03 | 0,82 |
| Gmeb2 | P58929 | 0,03 | 0,81 |
| Tlk1 | Q8C0V0 | 0,03 | 0,88 |
| Cic | Q924A2 | 0,02 | 0,88 |
| Batf | O35284 | 0,02 | 0,91 |
| Ing3 | Q8VEK6 | 0,02 | 0,88 |
| Foxo3 | Q9WVH4 | 0,02 | 0,94 |
| Ncoa3 | A2A468 | 0,02 | 0,90 |
| Begain | Q68EF6 | 0,02 | 0,95 |
| birA | P06709 | 0,01 | 0,95 |
| Vgll4 | Q80V24 | 0,01 | 0,96 |
| Cux1 | H3BK24 | 0,01 | 0,92 |
| Taf4a | E9QAP7 | 0,01 | 0,96 |
| Sap130 | A0A0R4J060 | 0,01 | 0,95 |
| Kdm3a | Q6PCM1 | 0,00 | 0,94 |
| Znf710 | Q3U288 | 0,00 | 0,99 |
| Tcf3 | A0A0R4J011 | 0,00 | 1,00 |
| Tcf12 | Q61286 | 0,00 | 0,99 |
| Cdc25c | P48967 | 0,00 | 0,99 |
| Smarcc1 | Q3UNN4 | -0,01 | 0,96 |
| Bcorl1 | A2AQH4 | -0,01 | 0,96 |
| Zfp266 | E9Q2S7 | -0,01 | 0,98 |
| Plagl2 | Q925T4 | -0,01 | 0,98 |
| Jmjd1c | G3UZM1 | -0,01 | 0,93 |
| Ncoa6 | Q5XJV5 | -0,01 | 0,89 |
| Myb | A0A087WPA7 | -0,01 | 0,96 |
| Foxp1 | D3Z6Q3 | -0,01 | 0,91 |
| Gps2 | Q921N8 | -0,01 | 0,85 |
| Nrip1 | Q8CBD1 | -0,02 | 0,88 |
| Zfp384 | E9QAR6 | -0,02 | 0,95 |
| Rreb1 | Q3UH06 | -0,02 | 0,81 |
| Dpf2 | Q61103 | -0,02 | 0,89 |
| Mafk | Q61827 | -0,02 | 0,89 |
| Maml2 | F6U238 | -0,02 | 0,87 |
| Zbtb7a | O88939 | -0,02 | 0,88 |
| Pias1 | O88907 | -0,02 | 0,87 |
| Rbbp4 | Q60972 | -0,02 | 0,71 |
| Slx4ip | Q9D7Y9 | -0,02 | 0,90 |
| Zhx2 | Q8C0C0 | -0,02 | 0,91 |
| Lcorl | Q3U285 | -0,02 | 0,86 |
| Pcgf1 | Q8R023 | -0,03 | 0,93 |
| Gltscr1 | F8VPZ9 | -0,03 | 0,77 |
| Kmt2c | Q8BRH4 | -0,03 | 0,87 |
| Zmym2 | Q9CU65 | -0,03 | 0,80 |
| Rlf | E9Q532 | -0,03 | 0,89 |
| Ncoa2 | Q61026 | -0,03 | 0,80 |
| Dlx1 | Q64317 | -0,03 | 0,87 |
| Rbbp7 | Q60973 | -0,03 | 0,84 |
| Atf2 | P16951 | -0,03 | 0,84 |
| Kdm3b | B9EKS2 | -0,03 | 0,48 |
| Nfatc1 | B5B2N4 | -0,03 | 0,86 |
| Kmt2d | A0A0A0MQ73 | -0,03 | 0,86 |
| Junb | P09450 | -0,04 | 0,88 |
| Foxc1 | Q61572 | -0,04 | 0,87 |
| Tfap4 | Q9JIZ5 | -0,04 | 0,88 |
| Tet3 | Q8BG87 | -0,04 | 0,80 |
| Cux1 | H3BJQ9 | -0,04 | 0,28 |
| Med12 | A2AGH8 | -0,05 | 0,82 |
| Fli1 | P26323 | -0,05 | 0,83 |
| Bcor | Q8CGN4 | -0,05 | 0,54 |
| Mier1 | Q5UAK0 | -0,05 | 0,86 |
| Zfp217 | Q3U0X6 | -0,05 | 0,70 |
| Ncor1 | Q5RIM6 | -0,05 | 0,62 |
| Ikzf1 | Q5SWT9 | -0,05 | 0,50 |
| Ebf1 | Q07802 | -0,05 | 0,63 |
| Smarca4 | Q3TKT4 | -0,06 | 0,82 |
| Mbd1 | A0A0R4J159 | -0,06 | 0,74 |
| Zeb2 | A0A0J9YV01 | -0,06 | 0,56 |
| Hivep2 | Q3UHF7 | -0,06 | 0,65 |
| Zbtb1 | Q91VL9 | -0,06 | 0,50 |
| Mafg | O54790 | -0,06 | 0,62 |
| Med1 | Q925J9 | -0,06 | 0,10 |
| _+_ | _Q9Z1D9_ | _-0,06_ | _0,84_ |
| Dpf1 | Q9QX66 | -0,07 | 0,83 |
| Smarcc2 | Q3UID0 | -0,07 | 0,71 |
| Phf21a | A2AHG4 | -0,07 | 0,53 |
| Zscan20 | B2KFW1 | -0,07 | 0,81 |
| Bcl11a | Q9QYE3 | -0,07 | 0,58 |
| Cux1 | H3BJL7 | -0,07 | 0,50 |
| Hdac2 | P70288 | -0,07 | 0,65 |
| Zfp646 | Q6NV66 | -0,07 | 0,67 |
| Zbtb21 | E9Q3R9 | -0,07 | 0,55 |
| Kansl1l | Q5DTI6 | -0,07 | 0,52 |
| Kat5 | Q8CHK4 | -0,08 | 0,73 |
| Lrrcc1 | Q69ZB0 | -0,08 | 0,44 |
| Smarce1 | O54941 | -0,08 | 0,66 |
| Znf608 | Q56A10 | -0,08 | 0,57 |
| Mybl2 | P48972 | -0,08 | 0,58 |
| Prr12 | E9PYL2 | -0,08 | 0,48 |
| Bcl9l | Q67FY2 | -0,08 | 0,69 |
| Jund | P15066 | -0,08 | 0,63 |
| Gse1 | Q3U3C9 | -0,08 | 0,43 |
| Homez | A0A0R4J108 | -0,08 | 0,82 |
| Bach2 | P97303 | -0,08 | 0,55 |
| Smarcd2 | Q99JR8 | -0,08 | 0,67 |
| Znf507 | Q6ZPY5 | -0,09 | 0,75 |
| Exo5 | Q9CXP9 | -0,09 | 0,71 |
| Ikzf3 | O08900 | -0,09 | 0,14 |
| Gltscr1l | Q8CHH5 | -0,09 | 0,69 |
| Znf609 | Q8BZ47 | -0,09 | 0,64 |
| Rfx7 | F8VPJ6 | -0,09 | 0,22 |
| Irf2 | P23906 | -0,09 | 0,68 |
| Maff | O54791 | -0,09 | 0,40 |
| Znf148 | Q61624 | -0,10 | 0,35 |
| Snw1 | A0A0B4J1E2 | -0,10 | 0,66 |
| Nfix | Q3TYK3 | -0,10 | 0,57 |
| Thyn1 | Q8BJZ7 | -0,10 | 0,60 |
| Mga | A2AWL7 | -0,10 | 0,47 |
| Zmym4 | A2A791 | -0,10 | 0,67 |
| Wiz | F6ZBR8 | -0,11 | 0,27 |
| Tdp2 | Q9JJX7 | -0,11 | 0,71 |
| Nfrkb | Q6PIJ4 | -0,11 | 0,27 |
| Pogz | D3YUW8 | -0,12 | 0,54 |
| Elk3 | P41971 | -0,12 | 0,53 |
| Etv6 | P97360 | -0,12 | 0,68 |
| Nsd1 | E9QAE4 | -0,12 | 0,23 |
| Tcf20 | Q9EPQ8 | -0,12 | 0,24 |
| Elmsan1 | E9Q2I4 | -0,12 | 0,45 |
| Zbtb33 | Q8BN78 | -0,13 | 0,52 |
| Cbx5 | Q61686 | -0,13 | 0,20 |
| Chd7 | A2AJK6 | -0,13 | 0,59 |
| Nfat5 | A0A1U6URG8 | -0,13 | 0,46 |
| Foxm1 | Q6P1H7 | -0,13 | 0,46 |
| Nrf1 | Q99K73 | -0,14 | 0,28 |
| Lin54 | Q571G4 | -0,14 | 0,48 |
| Zfp292 | Q9Z2U2 | -0,14 | 0,40 |
| Ankrd10 | Q99LW0 | -0,14 | 0,12 |
| Etv5 | Q9CXC9 | -0,14 | 0,37 |
| Ints10 | Q8K2A7 | -0,15 | 0,46 |
| Kdm6a | O70546 | -0,15 | 0,41 |
| Chd4 | Q6PDQ2 | -0,15 | 0,11 |
| Cebpa | P53566 | -0,15 | 0,20 |
| E2f8 | Q58FA4 | -0,15 | 0,31 |
| Mbtd1 | Q6P5G3 | -0,15 | 0,38 |
| Kansl1 | A2A5Y4 | -0,15 | 0,32 |
| Nab1 | Q61122 | -0,15 | 0,36 |
| Kmt2a | P55200 | -0,15 | 0,40 |
| Casz1 | Q9CWL2 | -0,15 | 0,52 |
| Nacc1 | Q7TSZ8 | -0,15 | 0,30 |
| Rcor1 | A0A140T8R7 | -0,15 | 0,60 |
| Ifi203 | E9QAN9 | -0,15 | 0,23 |
| Trps1 | G3UW90 | -0,15 | 0,24 |
| Phf14 | Q9D4H9 | -0,16 | 0,47 |
| Stag1 | F8WHU7 | -0,16 | 0,25 |
| Ep400 | F6R9G0 | -0,16 | 0,58 |
| Rere | Q80TZ9 | -0,16 | 0,46 |
| Kmt2e | Q3UG20 | -0,16 | 0,16 |
| Smarcb1 | Q9Z0H3 | -0,16 | 0,32 |
| Znf746 | Q3U133 | -0,16 | 0,25 |
| Arid5a | Q3U108 | -0,16 | 0,27 |
| Snapc4 | Q8BP86 | -0,17 | 0,53 |
| Asun | Q8QZV7 | -0,17 | 0,19 |
| Hira | Q61666 | -0,17 | 0,39 |
| Bcl11a | Q5STS9 | -0,17 | 0,21 |
| Mbd3 | Q9Z2D8 | -0,17 | 0,05 |
| Pax5 | Q02650 | -0,17 | 0,50 |
| Chd9 | Q8BYH8 | -0,17 | 0,62 |
| Taf8 | Q9EQH4 | -0,17 | 0,53 |
| Foxn2 | E9Q7L6 | -0,17 | 0,34 |
| Chd8 | Q09XV5 | -0,17 | 0,47 |
| Zmym5 | Q3U2E2 | -0,17 | 0,41 |
| Nr2c2 | G3X9W4 | -0,18 | 0,29 |
| Ino80d | Q66JY2 | -0,18 | 0,02 |
| Dot1l | Q6XZL8 | -0,18 | 0,38 |
| Mta1 | F8WHY8 | -0,18 | 0,13 |
| Foxk1 | P42128 | -0,18 | 0,37 |
| Skil | D3Z7C5 | -0,18 | 0,21 |
| Phf20l1 | Q8CCJ9 | -0,18 | 0,11 |
| Hcfc2 | G5E837 | -0,19 | 0,35 |
| Zfp652;Znf652 | F2Z443 | -0,19 | 0,38 |
| Ep400 | Q8CHI8 | -0,20 | 0,52 |
| Chd3 | B1AR17 | -0,20 | 0,25 |
| Bach1 | P97302 | -0,21 | 0,19 |
| L3mbtl2 | P59178 | -0,21 | 0,44 |
| Mta2 | Q9R190 | -0,21 | 0,07 |
| Mastl | Q8C0P0 | -0,21 | 0,15 |
| Chaf1b | Q9D0N7 | -0,22 | 0,20 |
| Zfp362 | B1ASA5 | -0,22 | 0,50 |
| Arid1a | E9QAQ7 | -0,23 | 0,14 |
| Yeats2 | Q3TUF7 | -0,23 | 0,46 |
| Zbtb9 | Q8CDC7 | -0,23 | 0,16 |
| Prpf18 | Q8BM39 | -0,23 | 0,39 |
| Creb1 | Q01147 | -0,24 | 0,24 |
| Baz2b | A2AUY4 | -0,24 | 0,38 |
| Arid1b | E9Q6R4 | -0,24 | 0,21 |
| Zfp219 | Q4VA98 | -0,24 | 0,16 |
| Epc2 | Q8C0I4 | -0,25 | 0,20 |
| Cecr2 | E9Q2Z1 | -0,25 | 0,24 |
| Paxip1 | Q6NZQ4 | -0,25 | 0,18 |
| Nipbl | Q6KCD5 | -0,25 | 0,34 |
| Mbd2 | Q9Z2E1 | -0,25 | 0,00 |
| Dpy30 | Q99LT0 | -0,26 | 0,54 |
| Chaf1a | Q9QWF0 | -0,27 | 0,02 |
| Aff3 | F8WJA7 | -0,27 | 0,27 |
| Csrp2bp | Q8CID0 | -0,27 | 0,31 |
| Mcm10 | Q0VBD2 | -0,29 | 0,12 |
| Znf281 | Q99LI5 | -0,29 | 0,19 |
| E2f7 | Q6S7F2 | -0,29 | 0,18 |
| Gatad2b | Q8VHR5 | -0,29 | 0,02 |
| Etv3 | Q8R4Z4 | -0,30 | 0,10 |
| Ldb1 | P70662 | -0,31 | 0,25 |
| Ahdc1 | Q6PAL7 | -0,31 | 0,32 |
| Zfp644 | E9Q624 | -0,31 | 0,28 |
| Ubn1 | Q4G0F8 | -0,31 | 0,00 |
| Gatad2a | Q8CHY6 | -0,32 | 0,08 |
| Zeb1 | Q64318 | -0,32 | 0,22 |
| Mnt | O08789 | -0,33 | 0,35 |
| Irf1 | P15314 | -0,33 | 0,25 |
| Ubn2 | Q80WC1 | -0,34 | 0,06 |
| Adnp | Q9Z103 | -0,34 | 0,12 |
| Stat4 | Q3V157 | -0,34 | 0,18 |
| Raver1 | Q9CW46 | -0,35 | 0,28 |
| Zfp715 | G3X9T1 | -0,35 | 0,16 |
| Znf865;Zfp865 | Q3U3I9 | -0,36 | 0,48 |
| Bend3 | Q6PAL0 | -0,37 | 0,01 |
| Pias4 | Q9JM05 | -0,38 | 0,14 |
| Fbxw11 | Q5SRY7 | -0,41 | 0,20 |
| Ncor1 | Q3UV08 | -0,41 | 0,06 |
| Sbno2 | Q7TNB8 | -0,41 | 0,12 |
| Arid5b | Q8BM75 | -0,47 | 0,10 |
| Orc3 | Q9JK30 | -0,57 | 0,10 |
| Parpbp | Q6IRT3 | -0,62 | 0,06 |
| Ebf3 | Q6NXL3 | -0,74 | 0,23 |

**Figure 3- source data 1.** List of the interactome of C/EBPa^R35A^ and C/EBPa^WT^ in B cells. Shown are log2-based differences in biotin labeling of the full length (p42) C/EBPa^R35A^protein over P42 C/EBPa^WT^ protein. P values indicate significance of differences in the corresponding interactions. See also legend of Figure 3B.
