# Supplementary figures and images for: Carm1-arginine methylation of the transcription factor C/EBPα regulates transdifferentiation velocity
Source: eLife. 2023 Jun 27;12:e83951. doi: 10.7554/eLife.83951 (PMC10299824; doi:10.7554/eLife.83951)

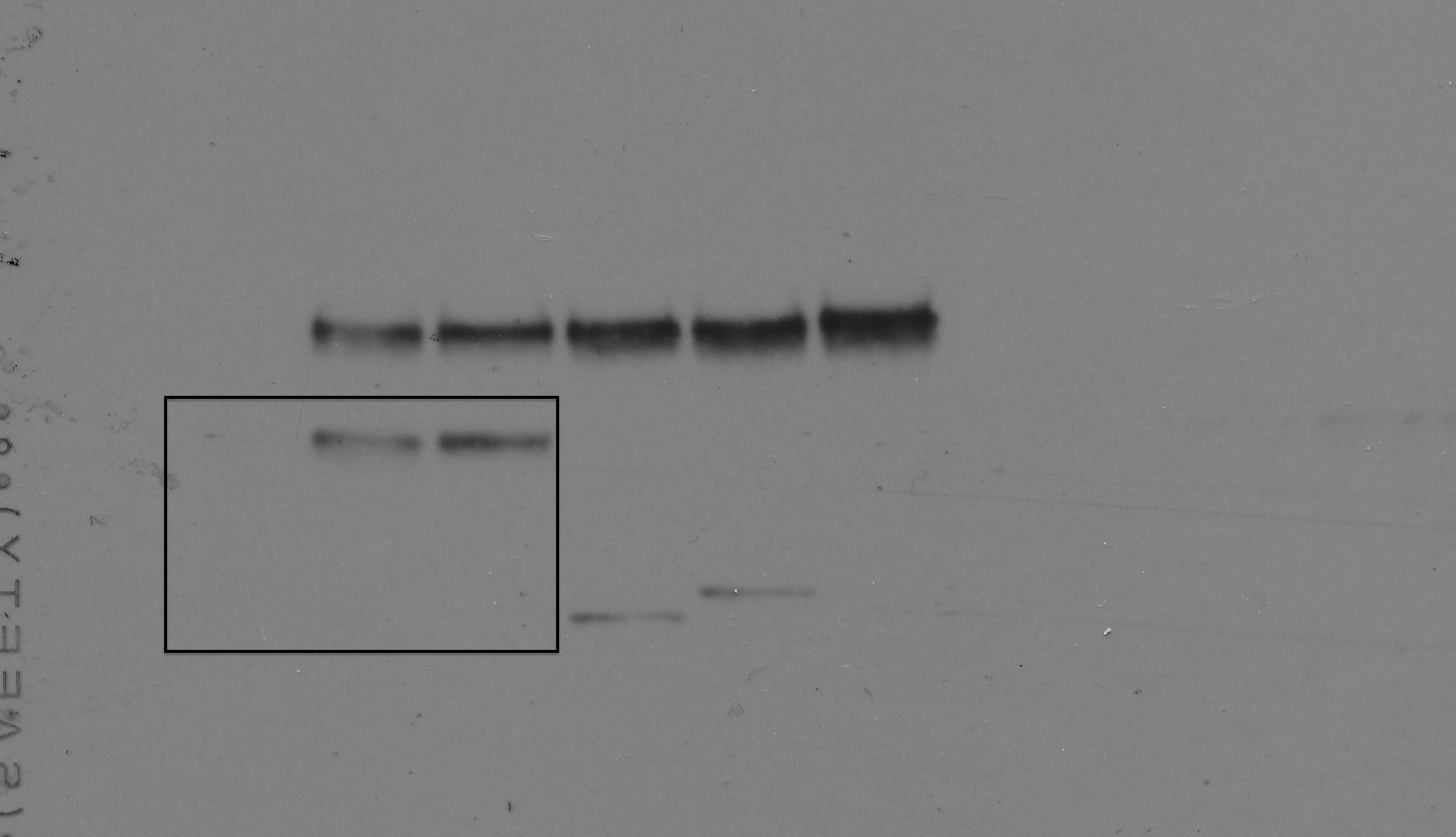

Supplement: Figure 3—source data 3. [file elife-83951-fig3-data3.zip › Figure 3C coIP CEBPA IB anti-FLAG marked.tif]

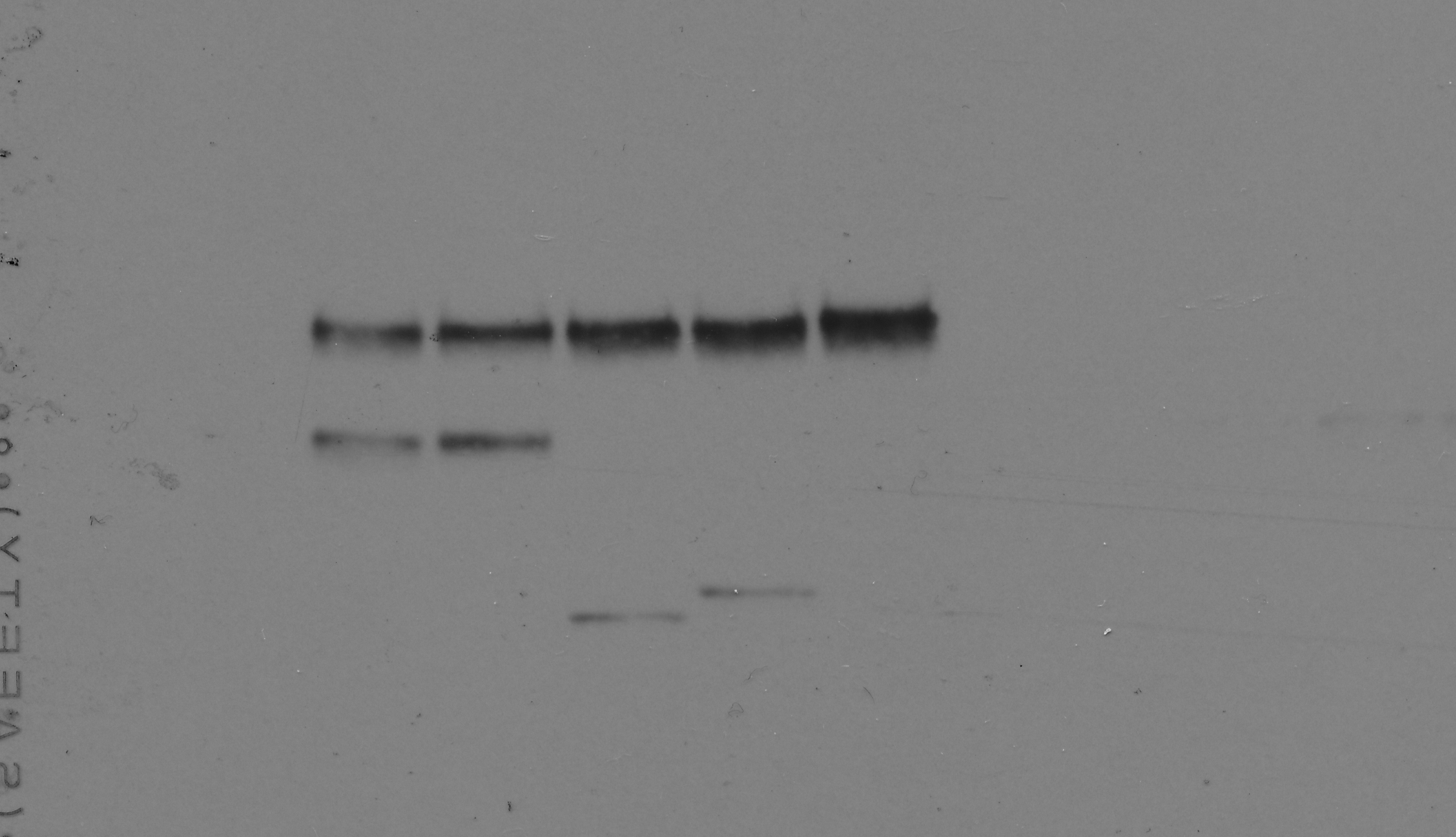

Supplement: Figure 3—source data 3. [file elife-83951-fig3-data3.zip › Figure 3C coIP CEBPA IB anti-FLAG source.tif]

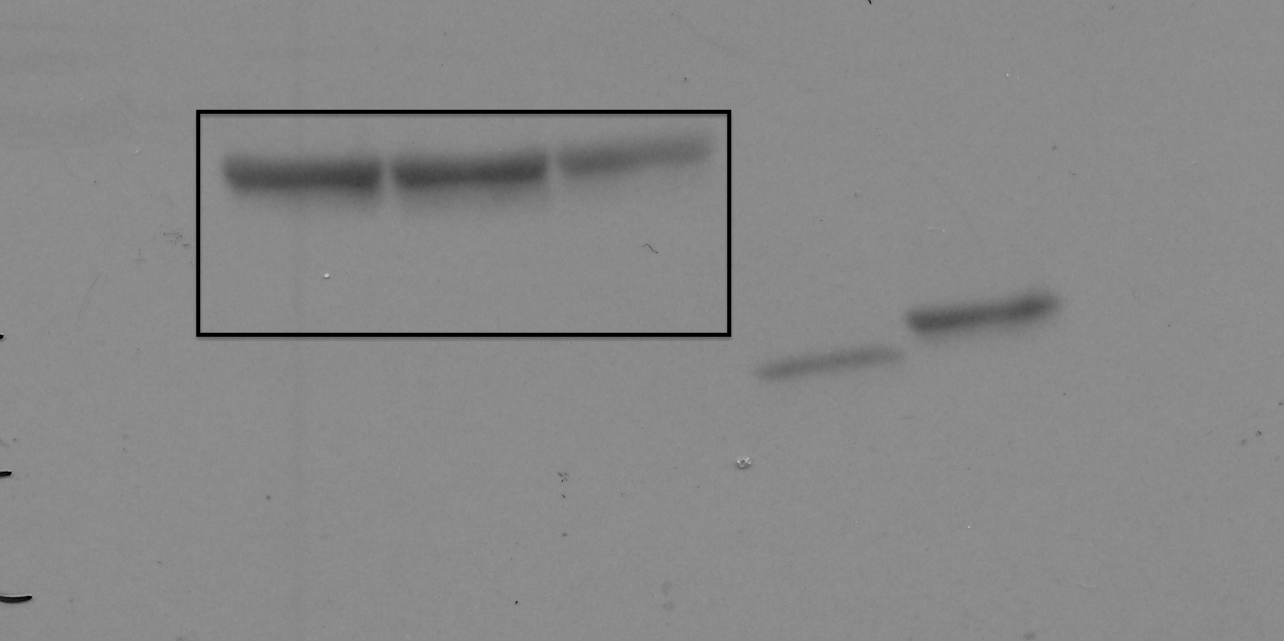

Supplement: Figure 3—source data 3. [file elife-83951-fig3-data3.zip › Figure 3C exp. ctrl. CEBPA, IB anti-Flag marked.tif]

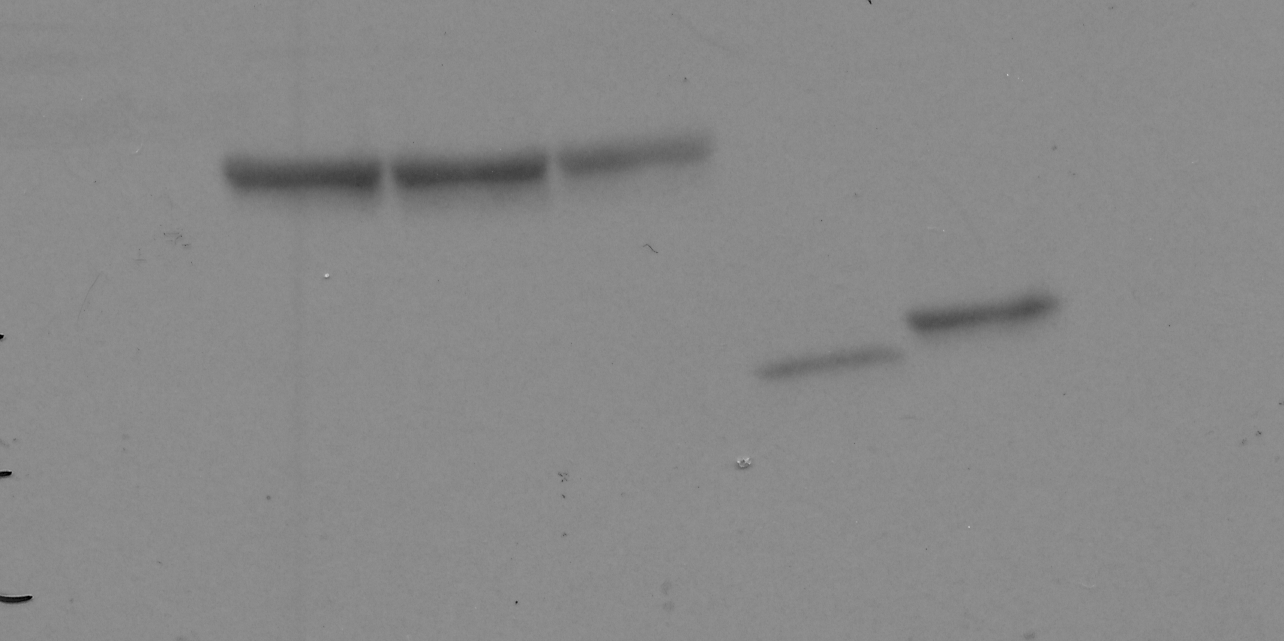

Supplement: Figure 3—source data 3. [file elife-83951-fig3-data3.zip › Figure 3C exp. ctrl. CEBPA, IB anti-Flag source.tif]

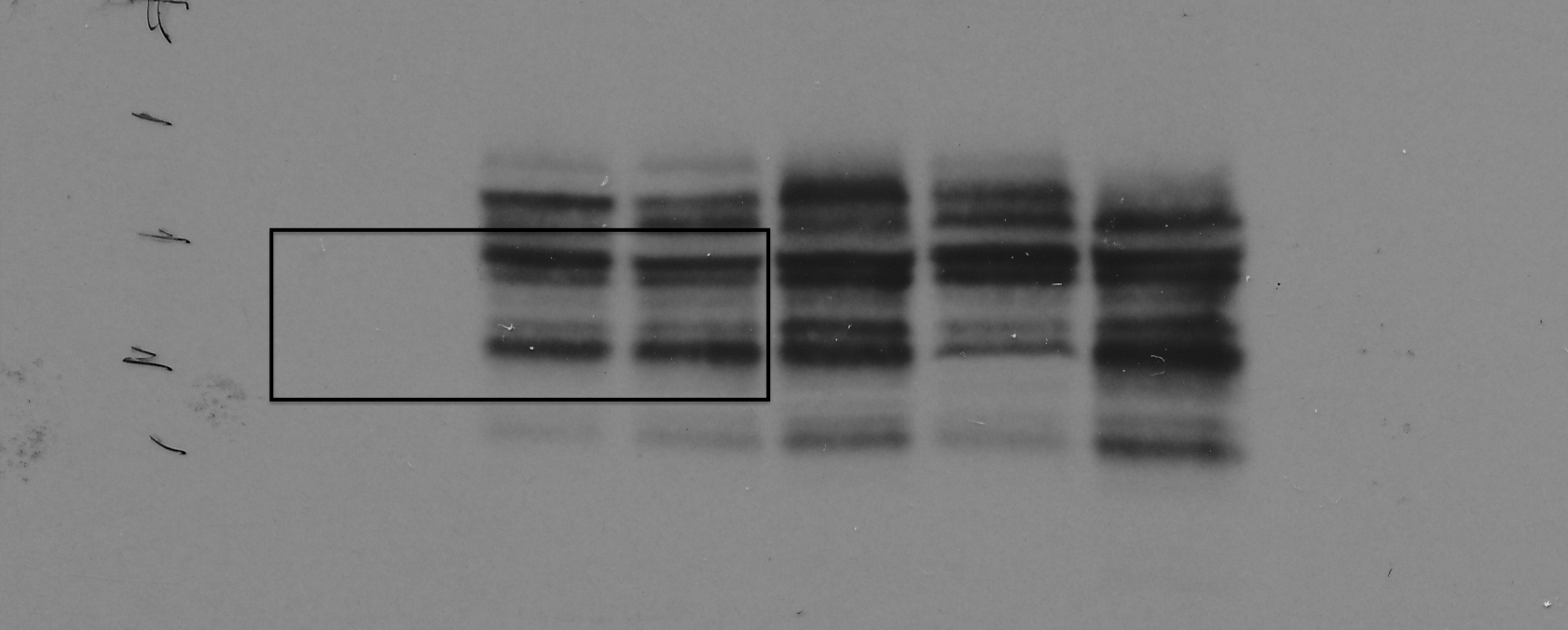

Supplement: Figure 3—source data 3. [file elife-83951-fig3-data3.zip › Figure 3C IP antiHA,IB antiPU.1 marked.tiff]

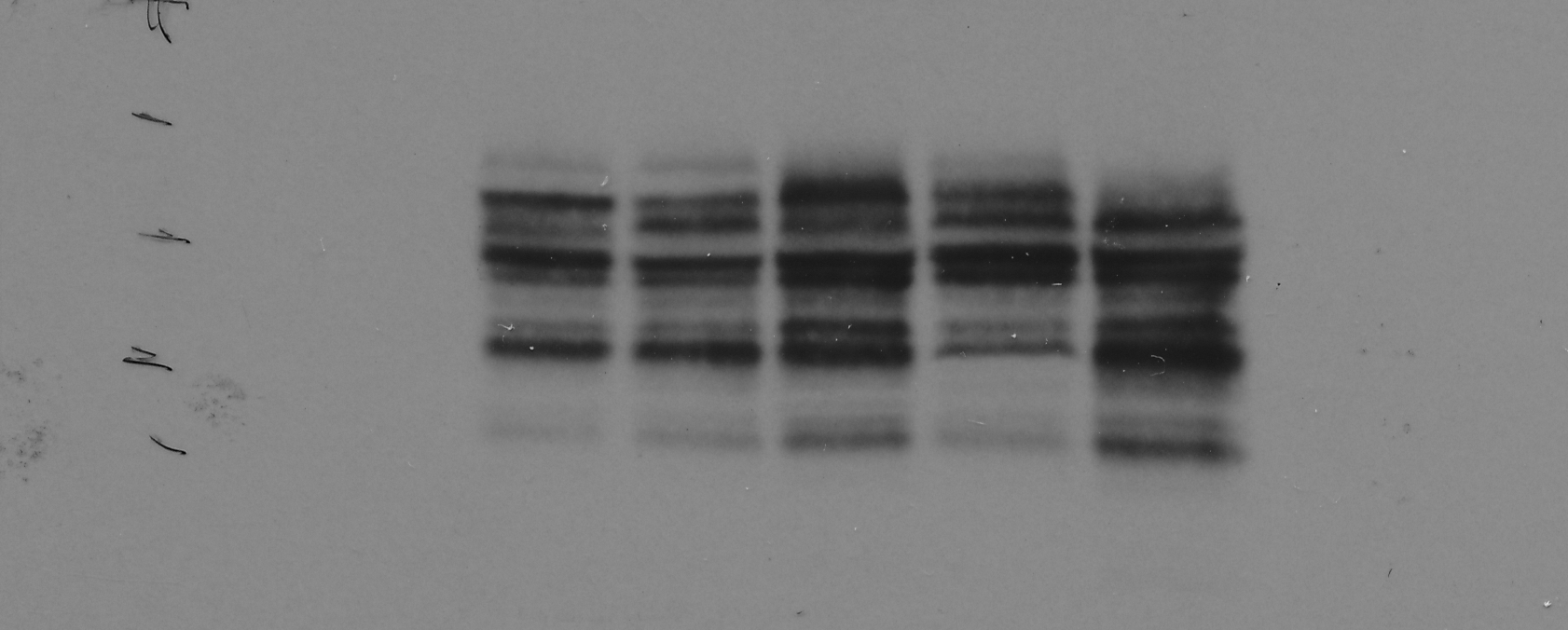

Supplement: Figure 3—source data 3. [file elife-83951-fig3-data3.zip › Figure 3C IP antiHA,IB antiPU.1 source.tiff]

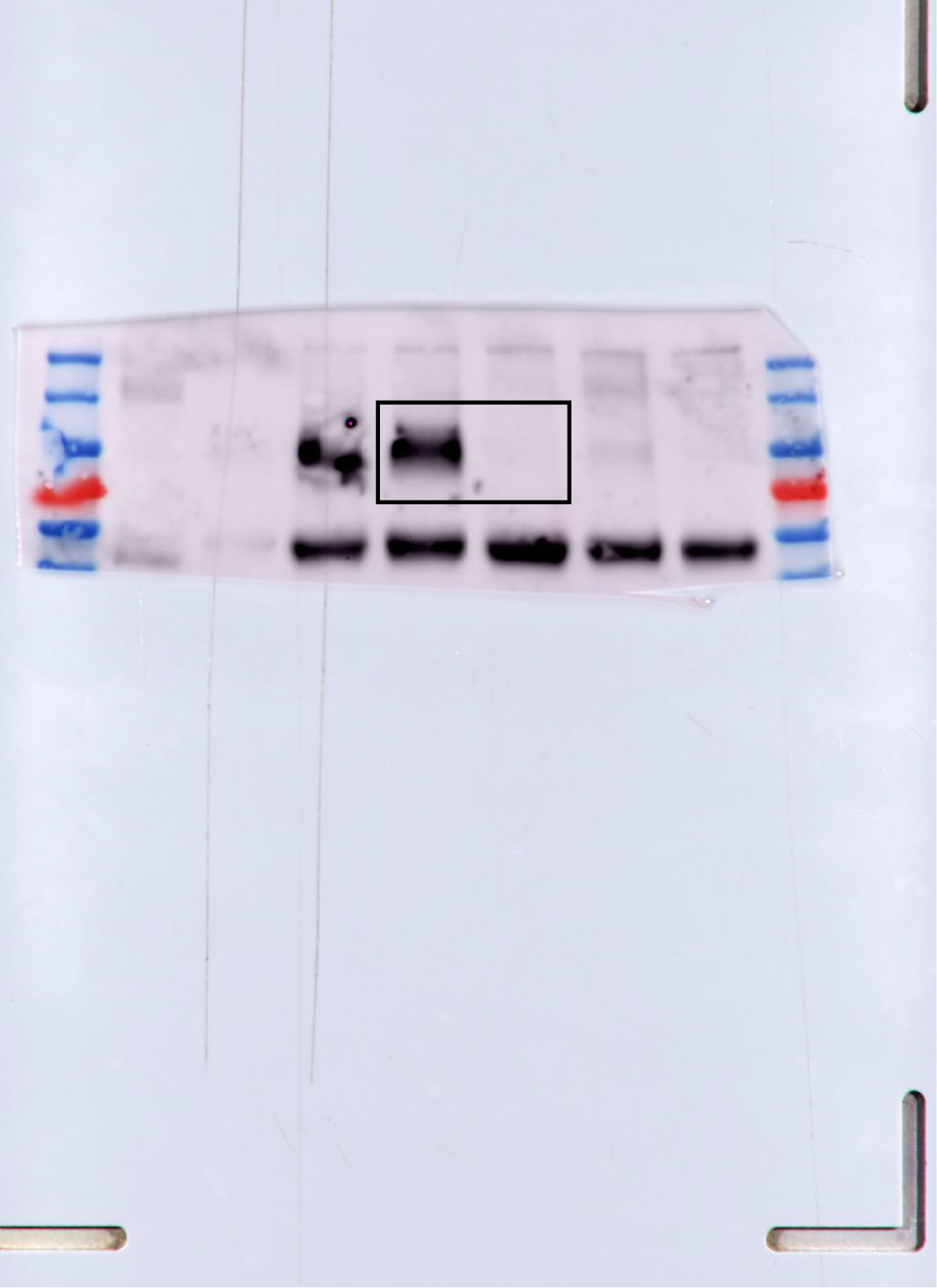

Supplement: Figure 7—source data 1. [file elife-83951-fig7-data1.zip › Figure_5A_Source/Fig5A marked.tif]

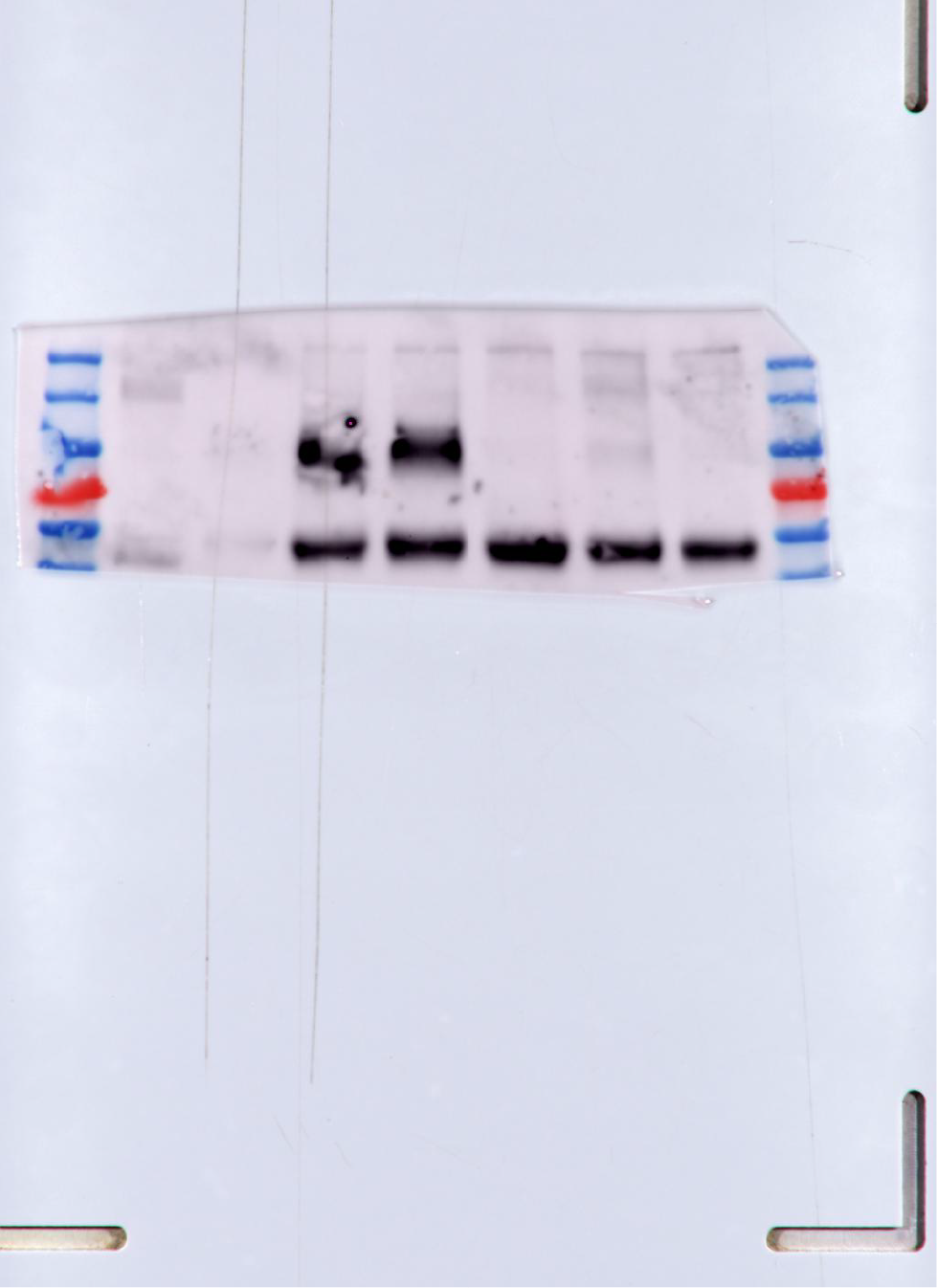

Supplement: Figure 7—source data 1. [file elife-83951-fig7-data1.zip › Figure_5A_Source/Fig5A source.tif]

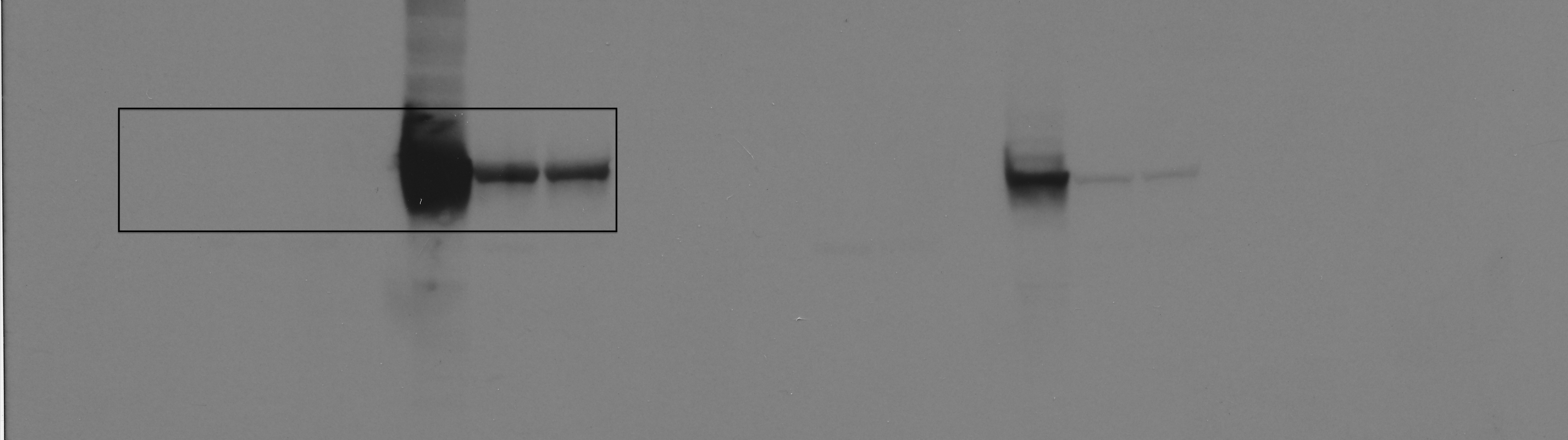

Supplement: Figure 7—source data 2. [file elife-83951-fig7-data2.zip › Figure 5B exp.ctrl. Carm1, IB anti-HA marked.tif]

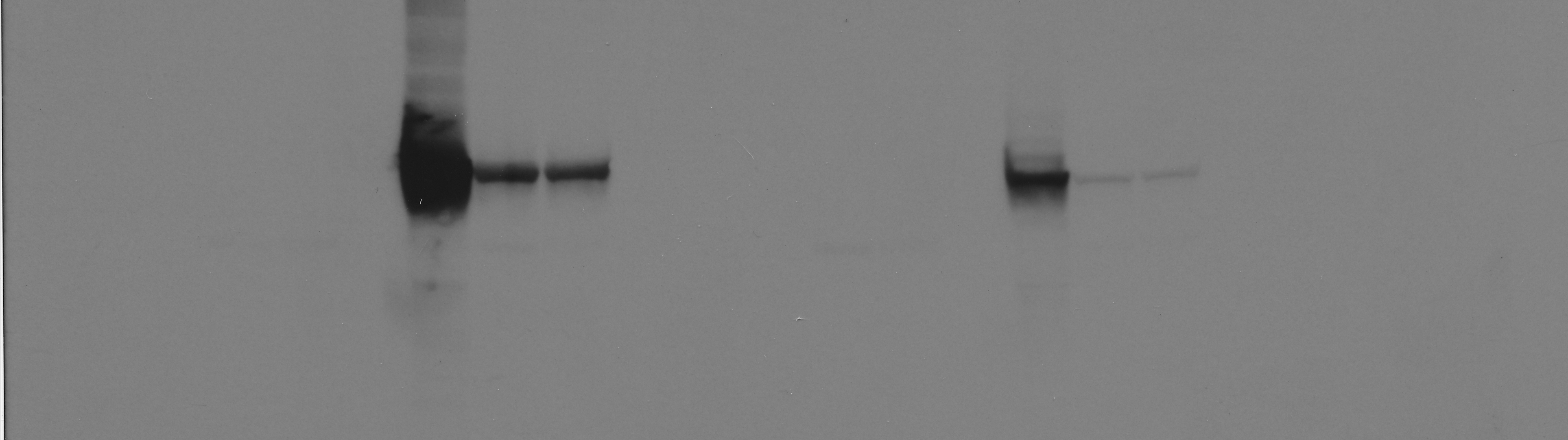

Supplement: Figure 7—source data 2. [file elife-83951-fig7-data2.zip › Figure 5B exp.ctrl. Carm1, IB anti-HA source.tif]

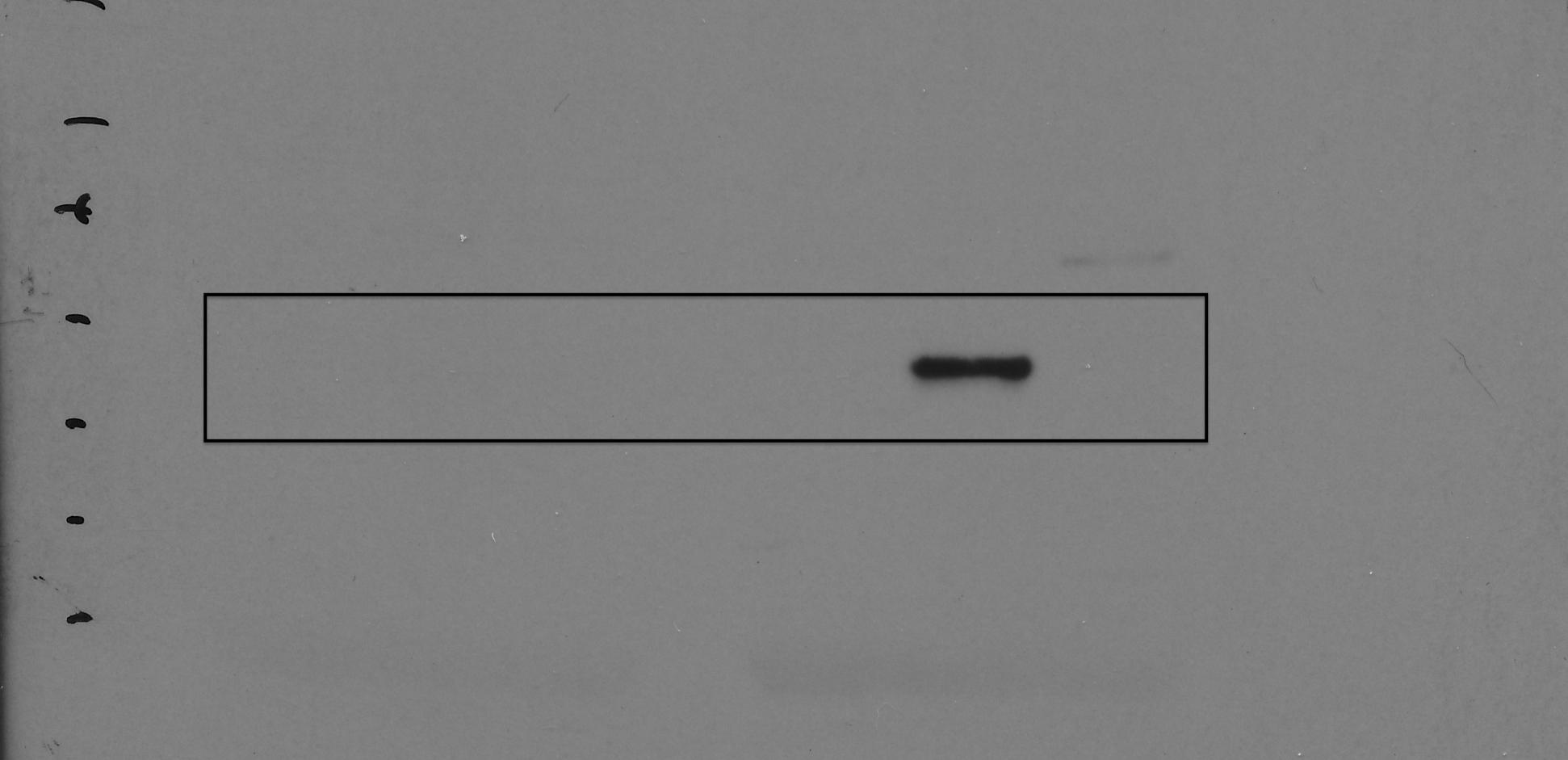

Supplement: Figure 7—source data 2. [file elife-83951-fig7-data2.zip › Figure 5B IP CEBPA, IB anti-DMA marked.tif]

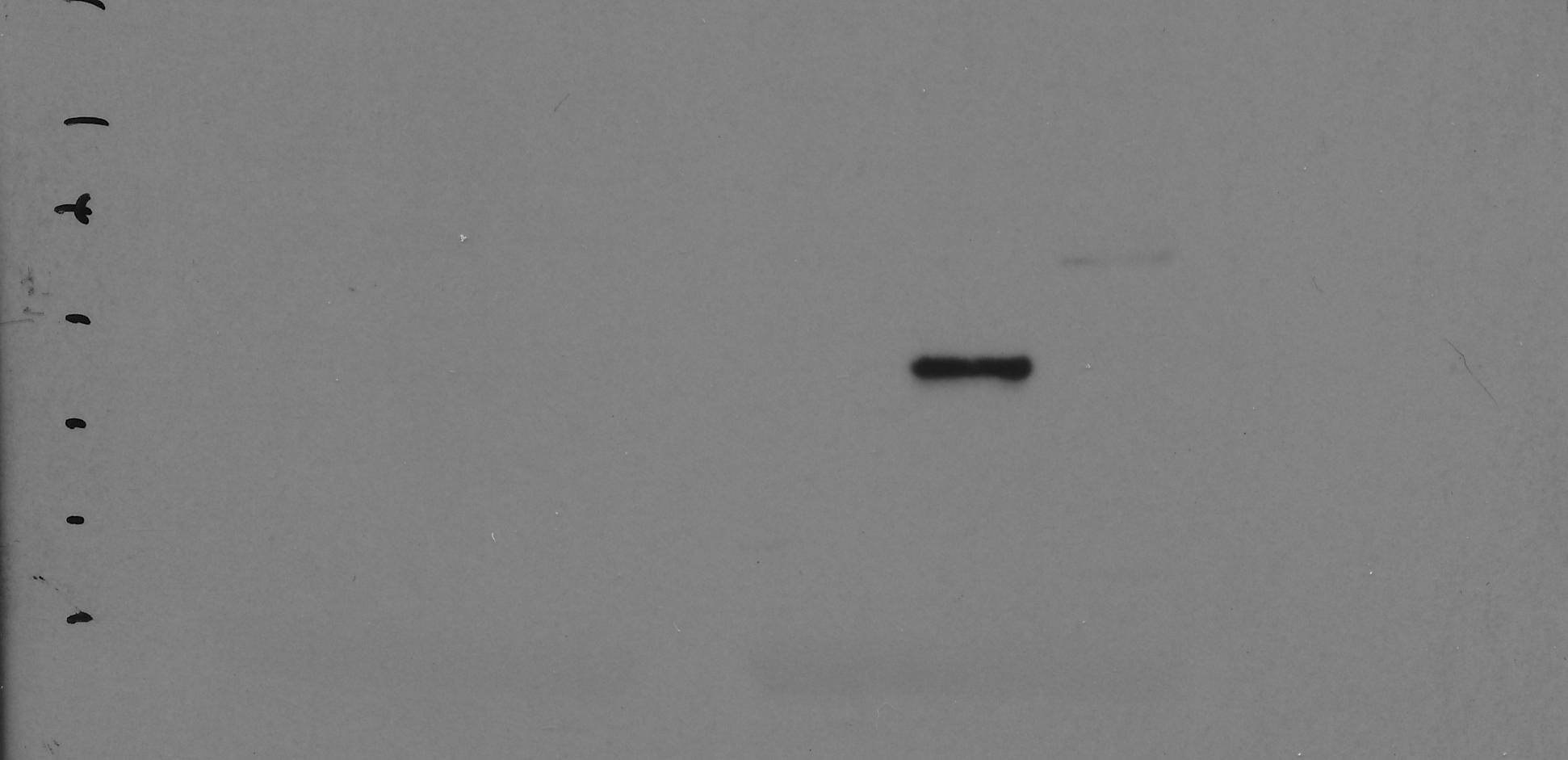

Supplement: Figure 7—source data 2. [file elife-83951-fig7-data2.zip › Figure 5B IP CEBPA, IB anti-DMA source .tif]

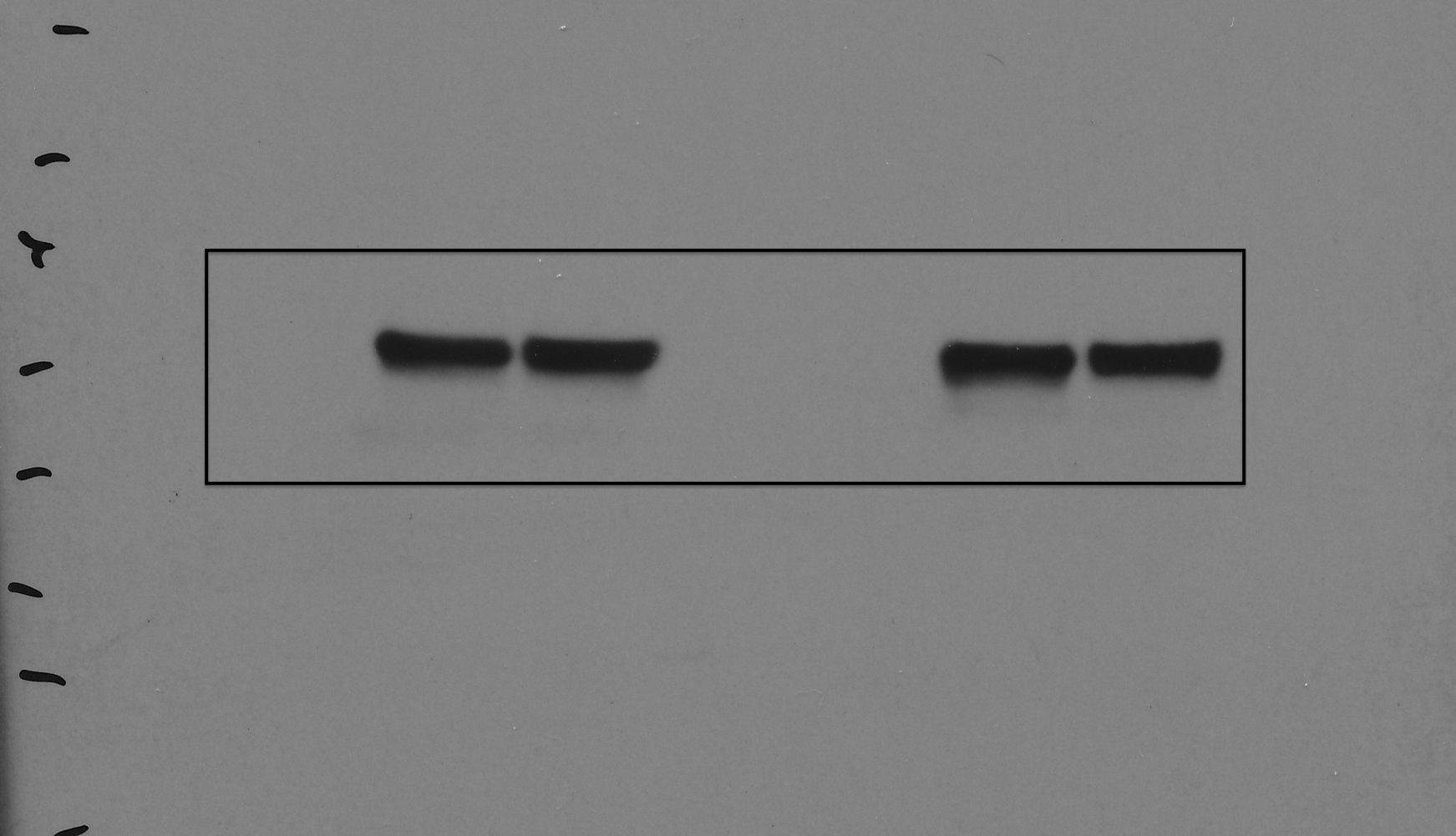

Supplement: Figure 7—source data 2. [file elife-83951-fig7-data2.zip › Figure 5B IP CEBPA, IB anti-Flag marked.tif]

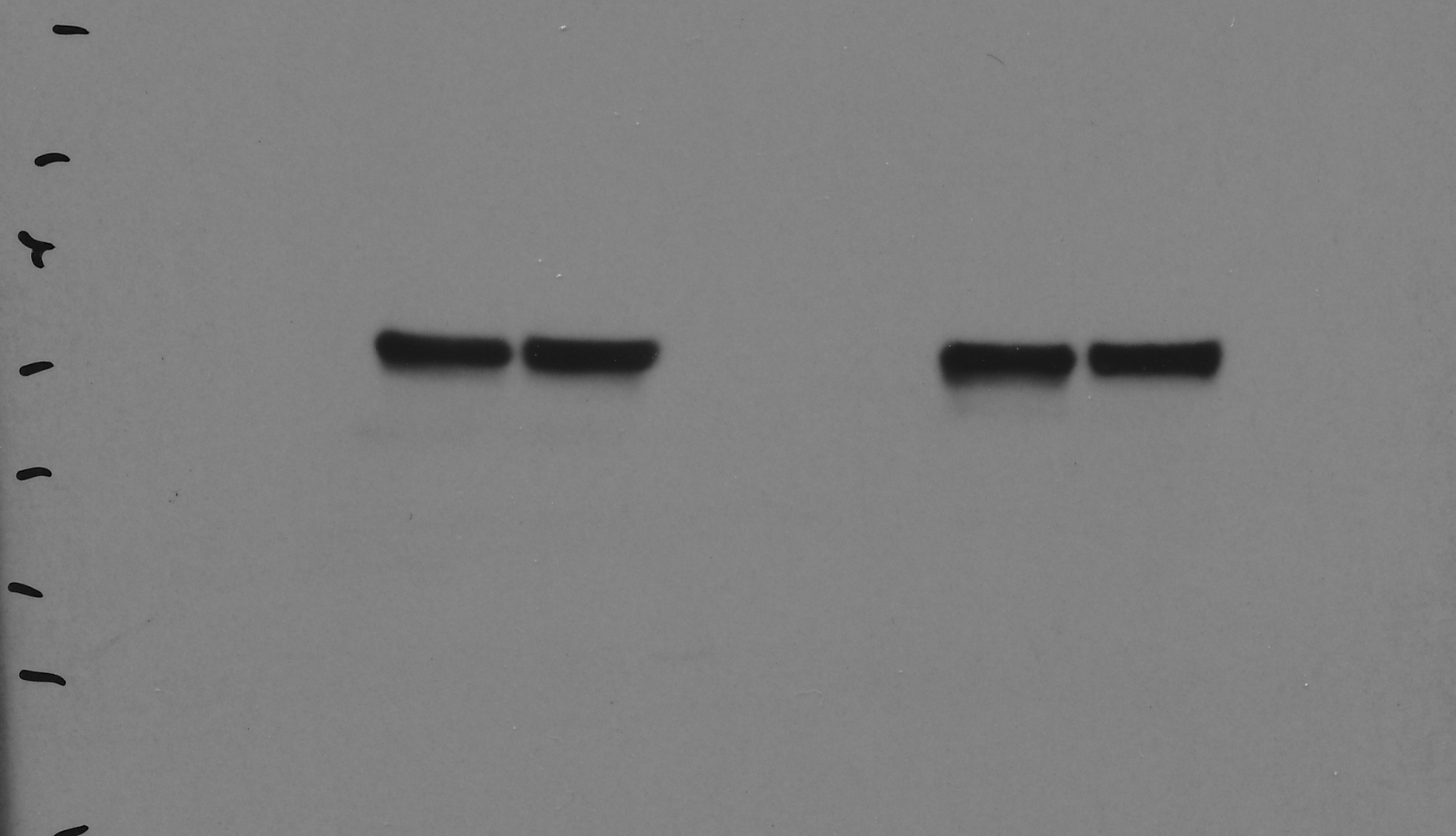

Supplement: Figure 7—source data 2. [file elife-83951-fig7-data2.zip › Figure 5B IP CEBPA, IB anti-Flag source.tif]

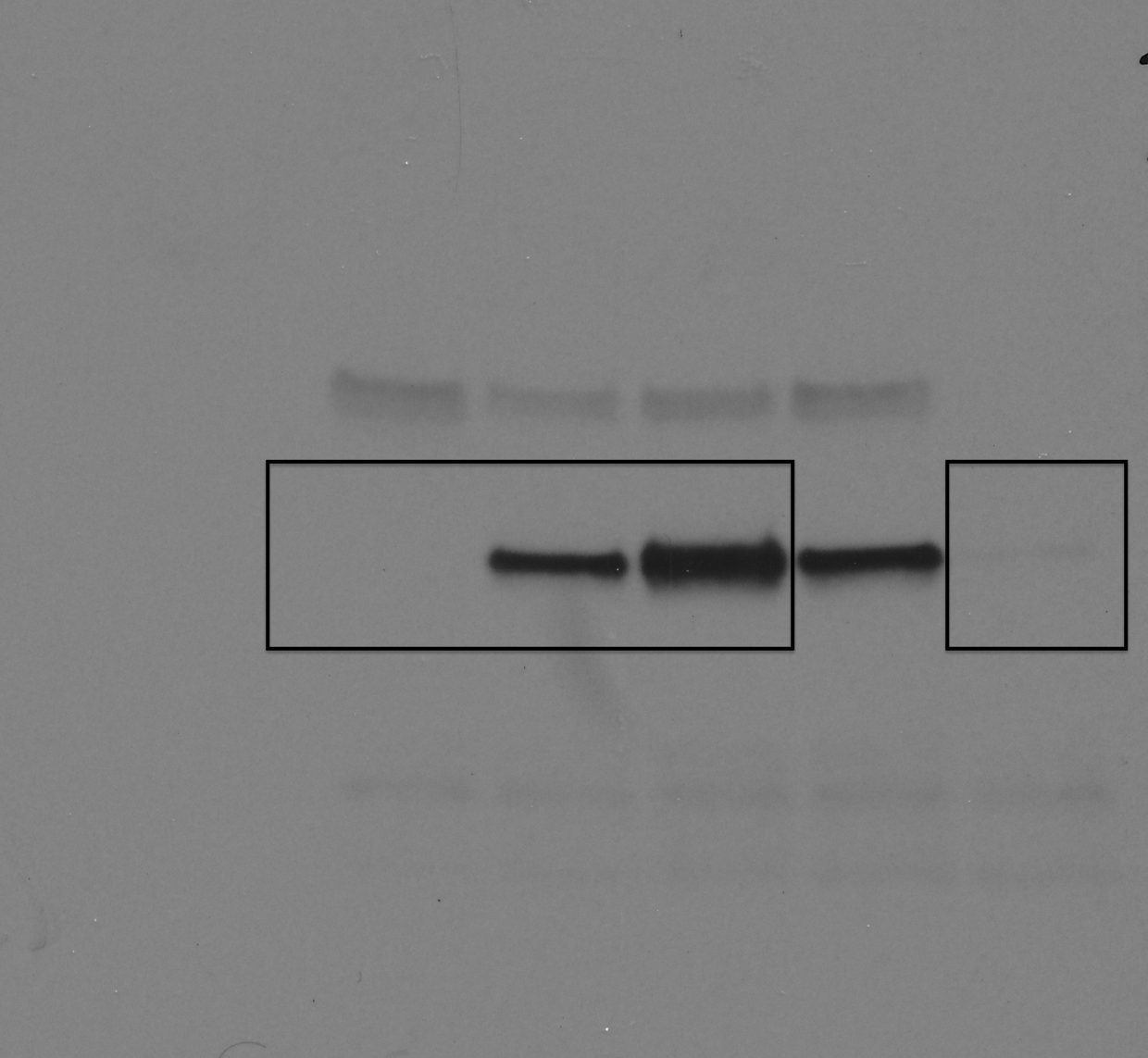

Supplement: Figure 7—source data 3. [file elife-83951-fig7-data3.zip › Figure 5C coIP CEBPA, IB anti-Flag marked.tif]

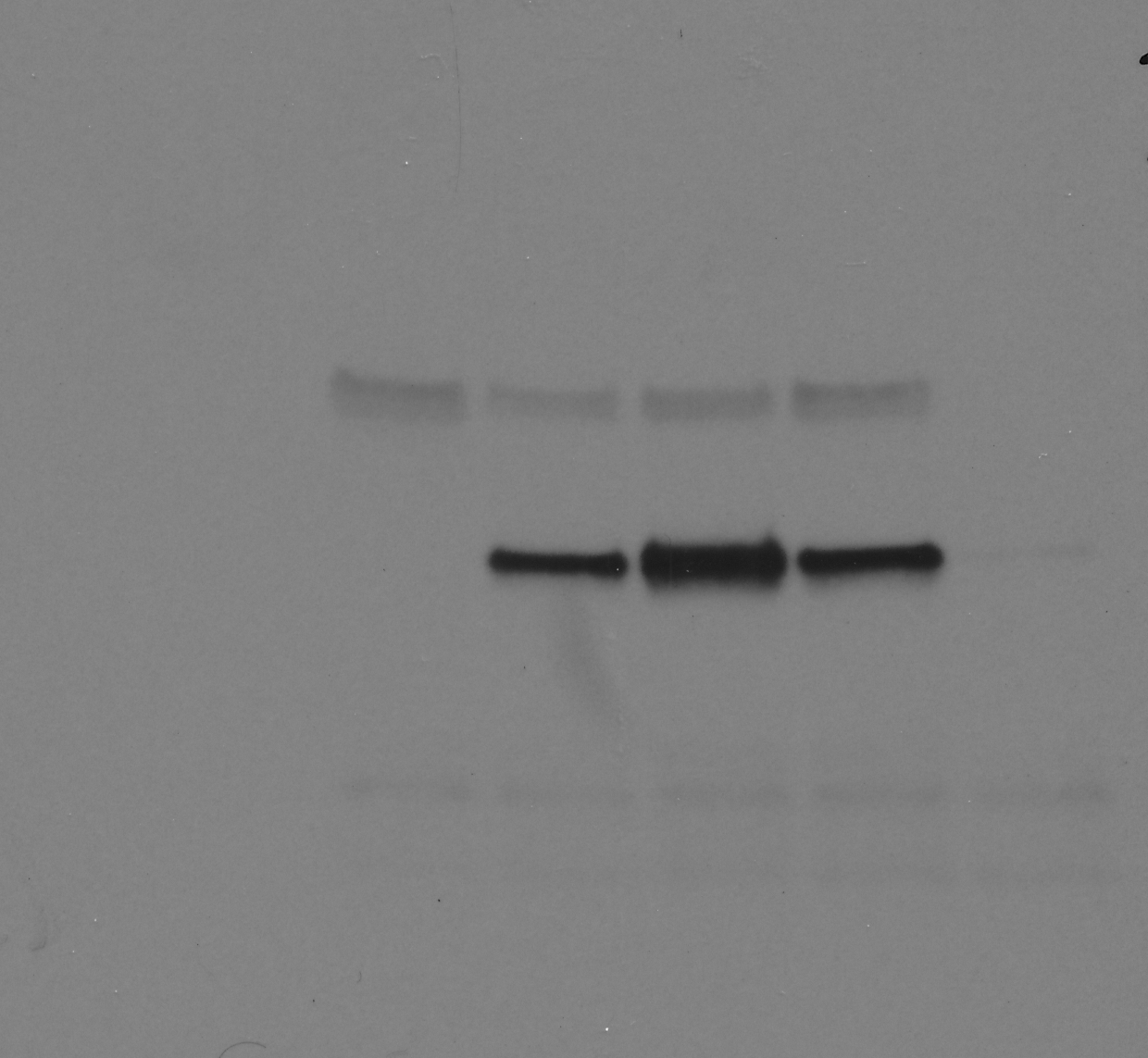

Supplement: Figure 7—source data 3. [file elife-83951-fig7-data3.zip › Figure 5C coIP CEBPA, IB anti-Flag source.tif]

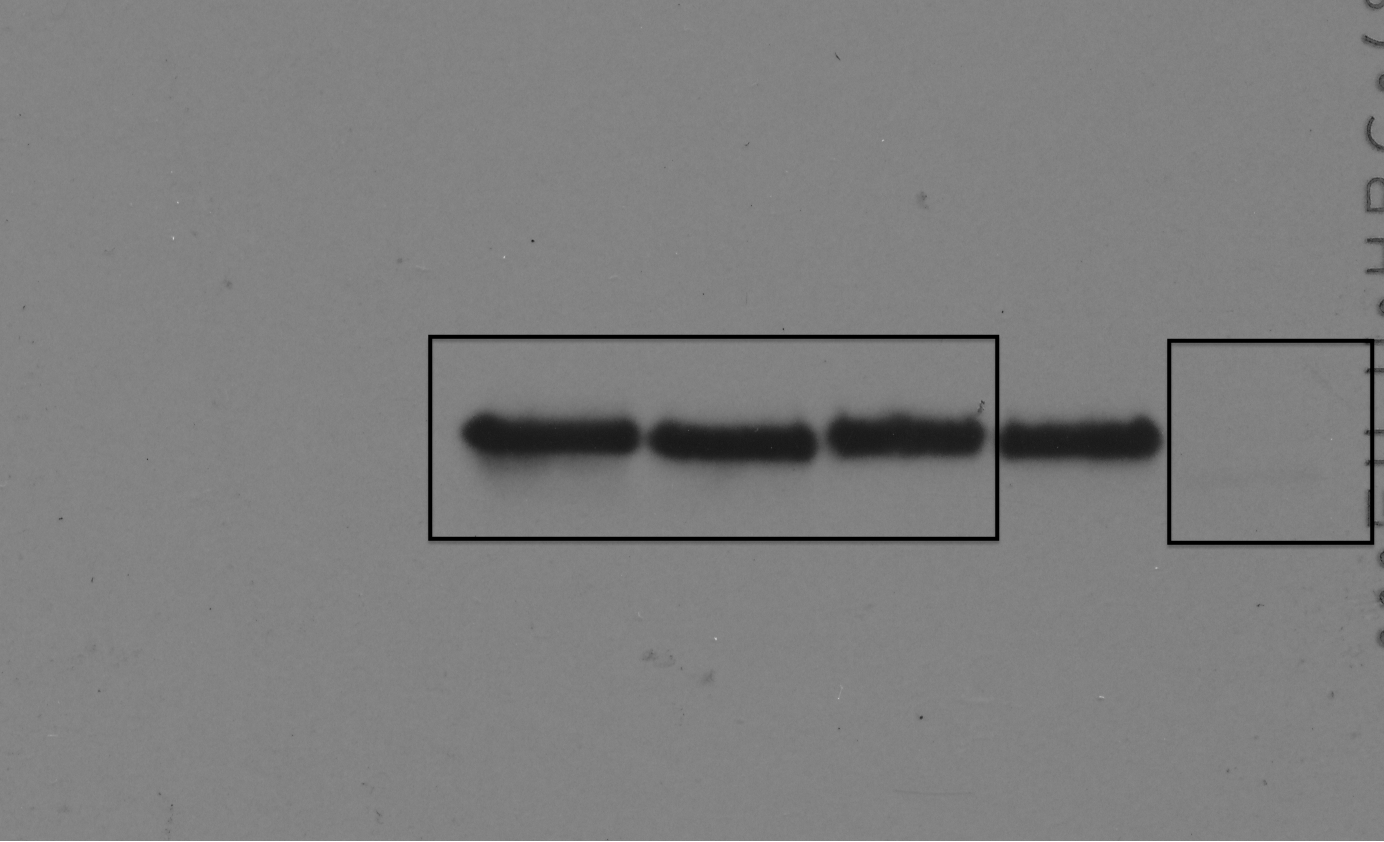

Supplement: Figure 7—source data 3. [file elife-83951-fig7-data3.zip › Figure 5C exp. ctrl. CEBPA IB anti-Flag marked.tif]

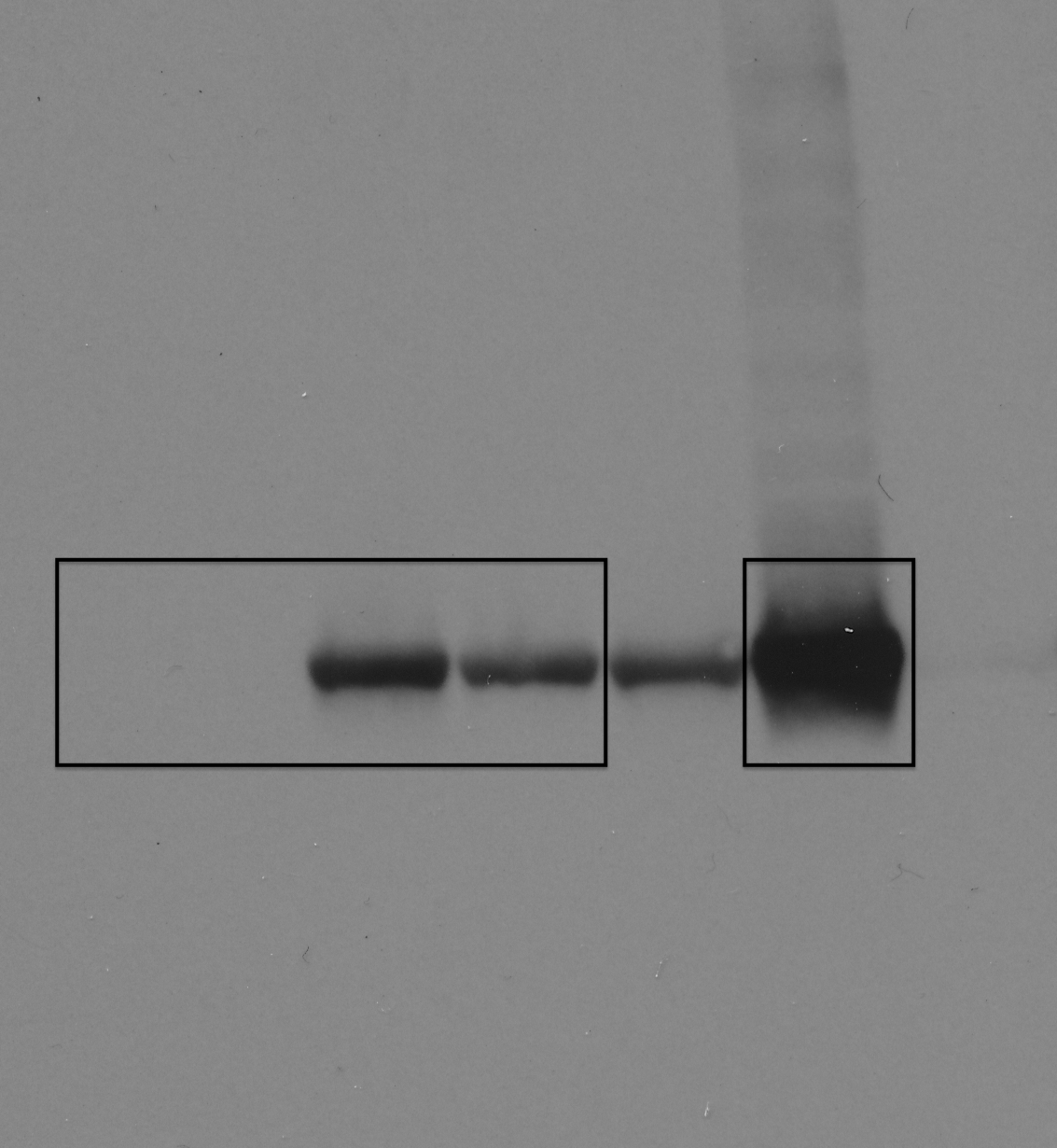

Supplement: Figure 7—source data 3. [file elife-83951-fig7-data3.zip › Figure 5C IP Carm1-HA IB anti-HA marked.tiff]

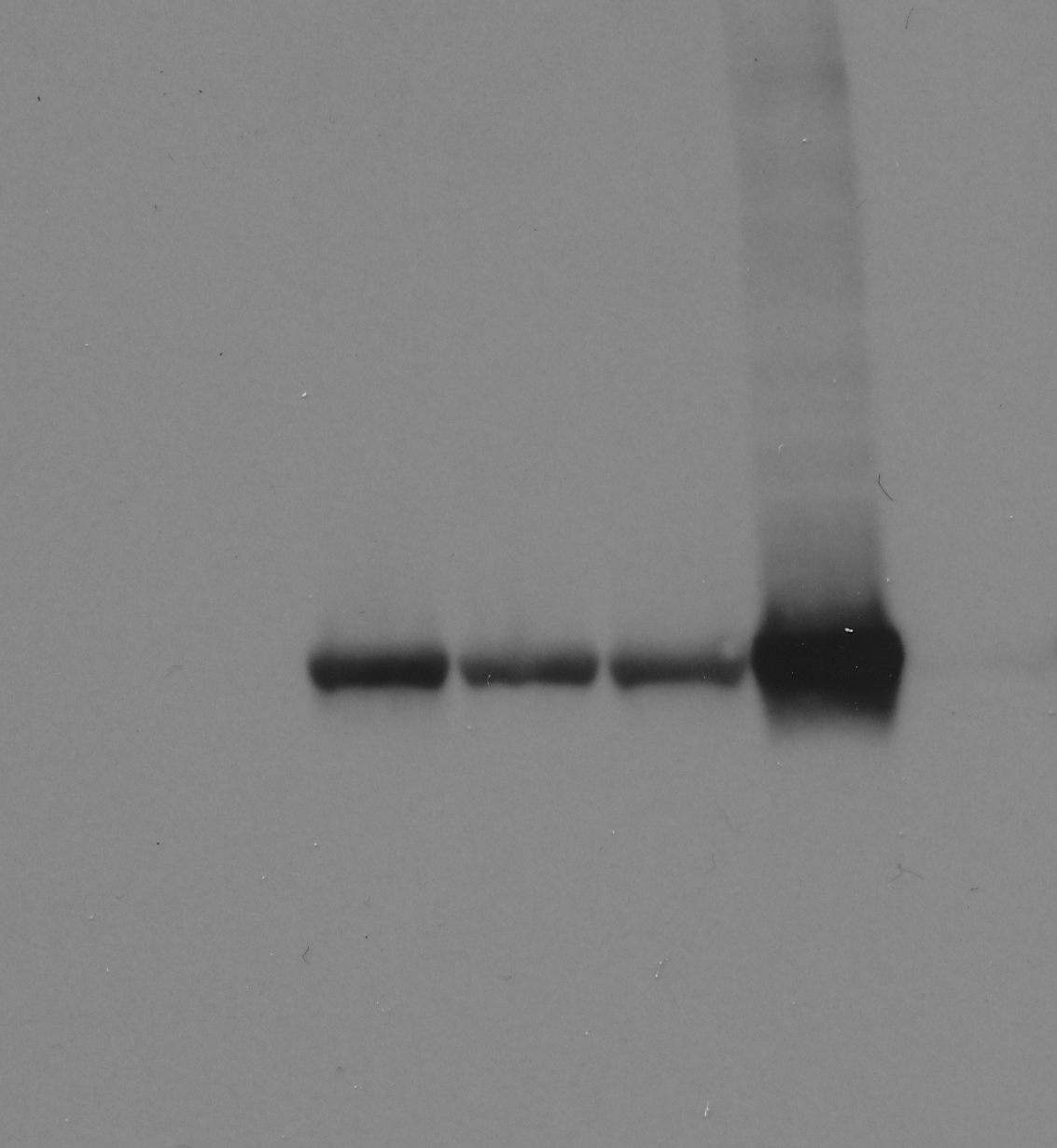

Supplement: Figure 7—source data 3. [file elife-83951-fig7-data3.zip › Figure 5C IP Carm1-HA IB anti-HA source.tiff]

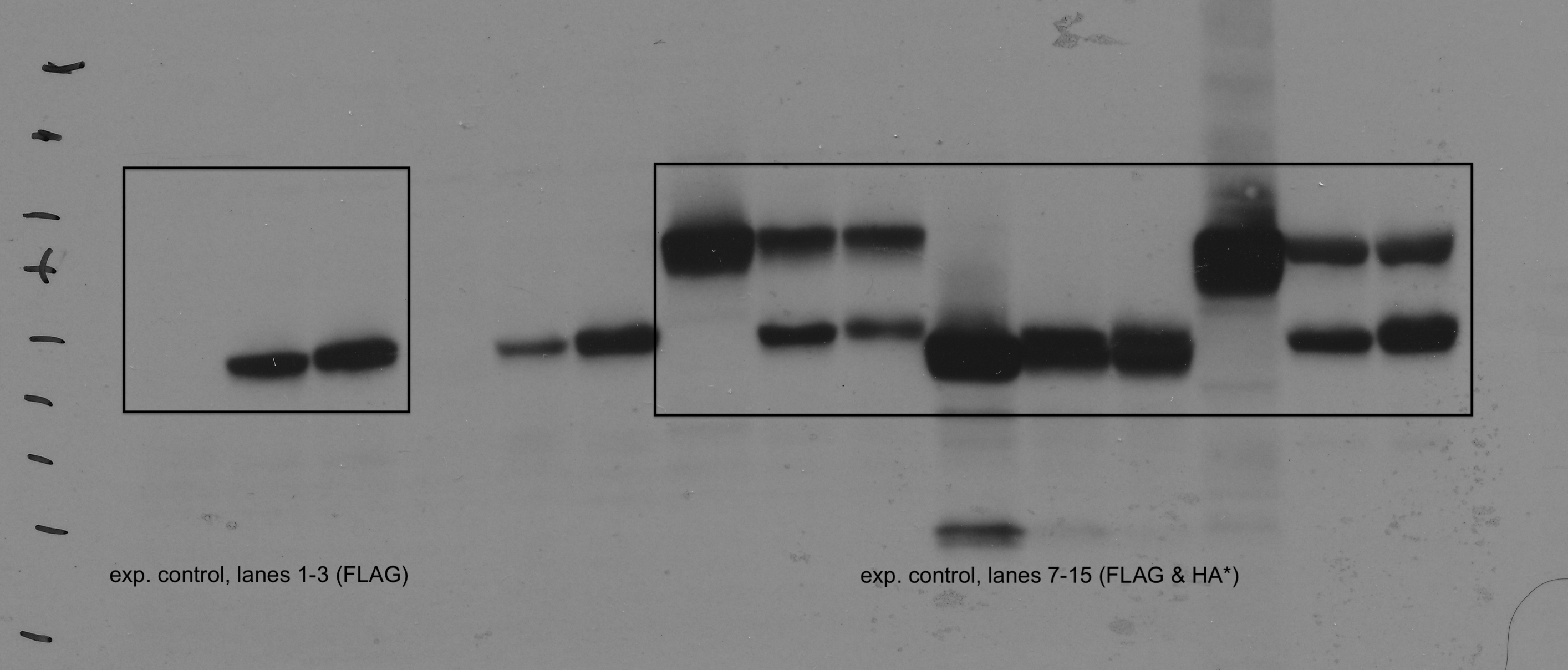

Supplement: Figure 7—figure supplement 1—source data 1. [file elife-83951-fig7-figsupp1-data1.zip › Figure S5 exp.ctrl CEBPA, -:+ PRMTs, IB antiFLAG:HA marked.tif]

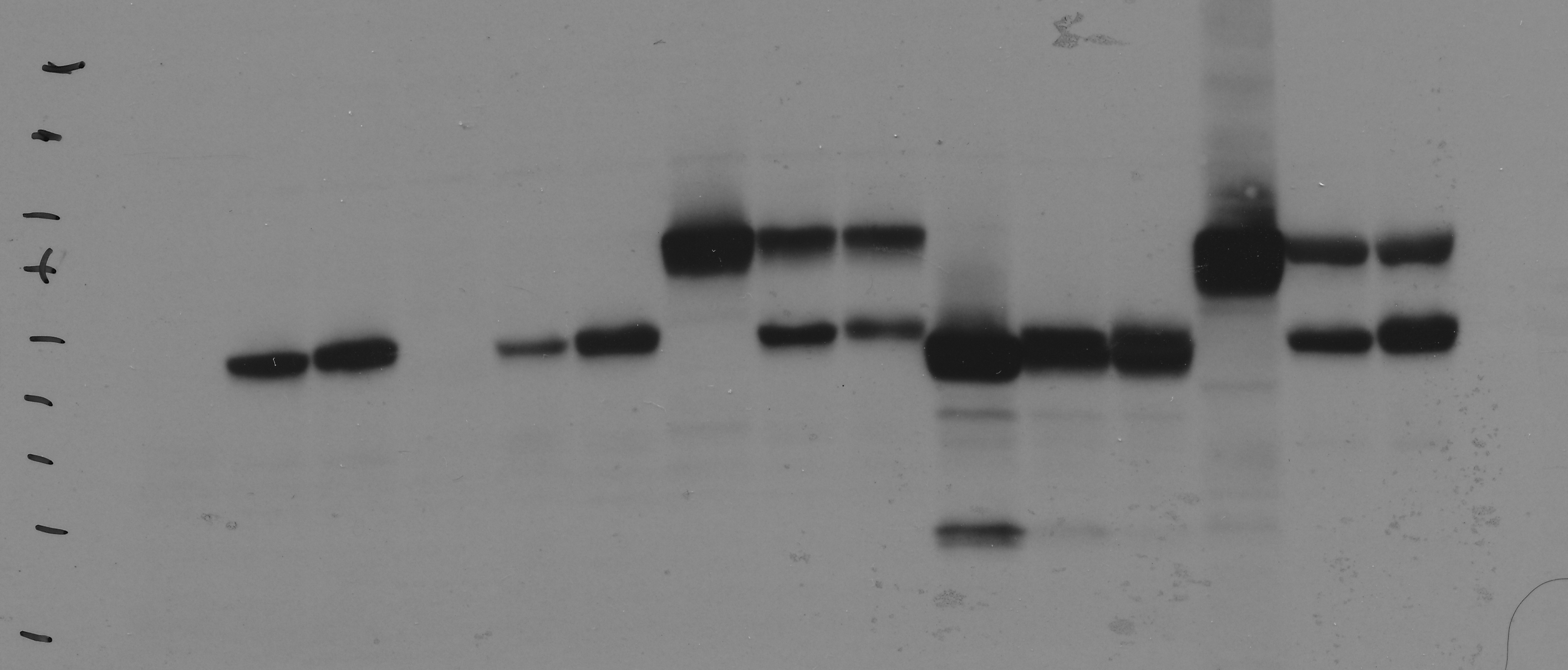

Supplement: Figure 7—figure supplement 1—source data 1. [file elife-83951-fig7-figsupp1-data1.zip › Figure S5 exp.ctrl CEBPA, -:+ PRMTs, IB antiFLAG:HA source.tif]

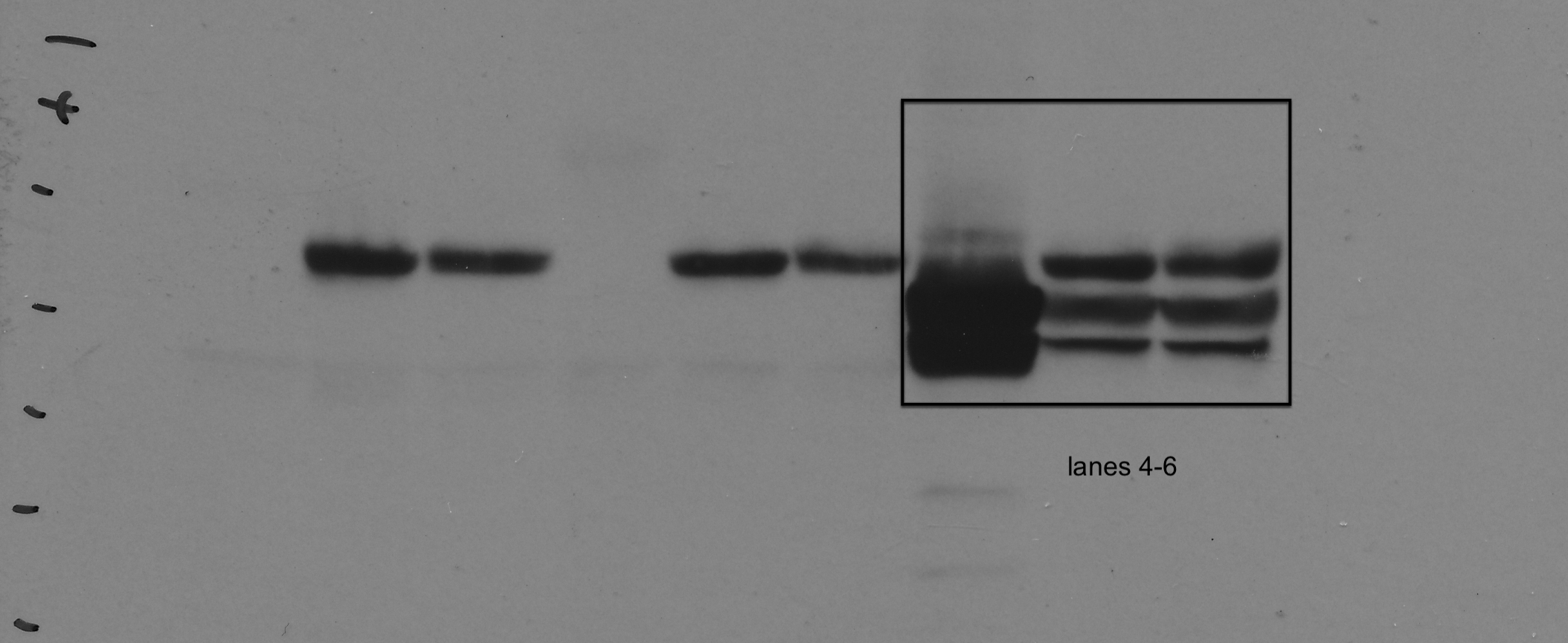

Supplement: Figure 7—figure supplement 1—source data 1. [file elife-83951-fig7-figsupp1-data1.zip › Figure S5 exp.ctrl. PRMT1, IB antiFLAG:HA marked.tif]

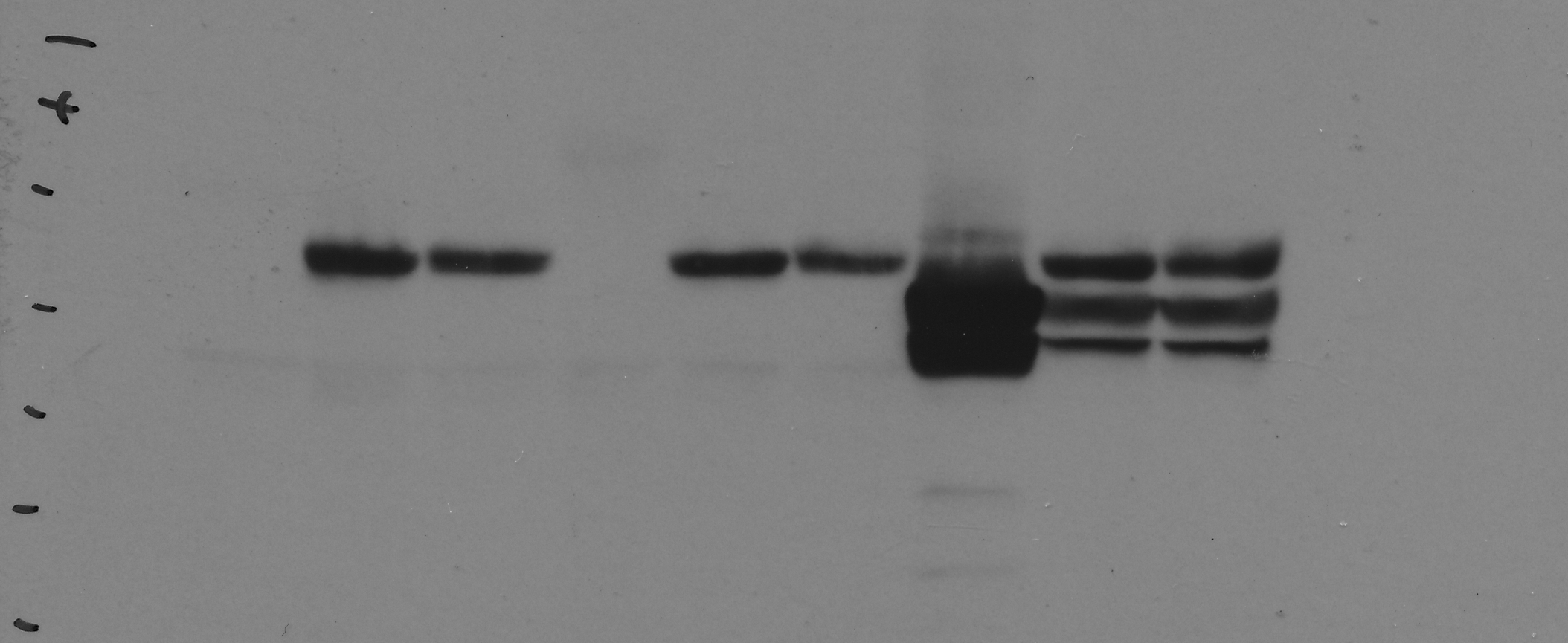

Supplement: Figure 7—figure supplement 1—source data 1. [file elife-83951-fig7-figsupp1-data1.zip › Figure S5 exp.ctrl. PRMT1, IB antiFLAG:HA source.tif]

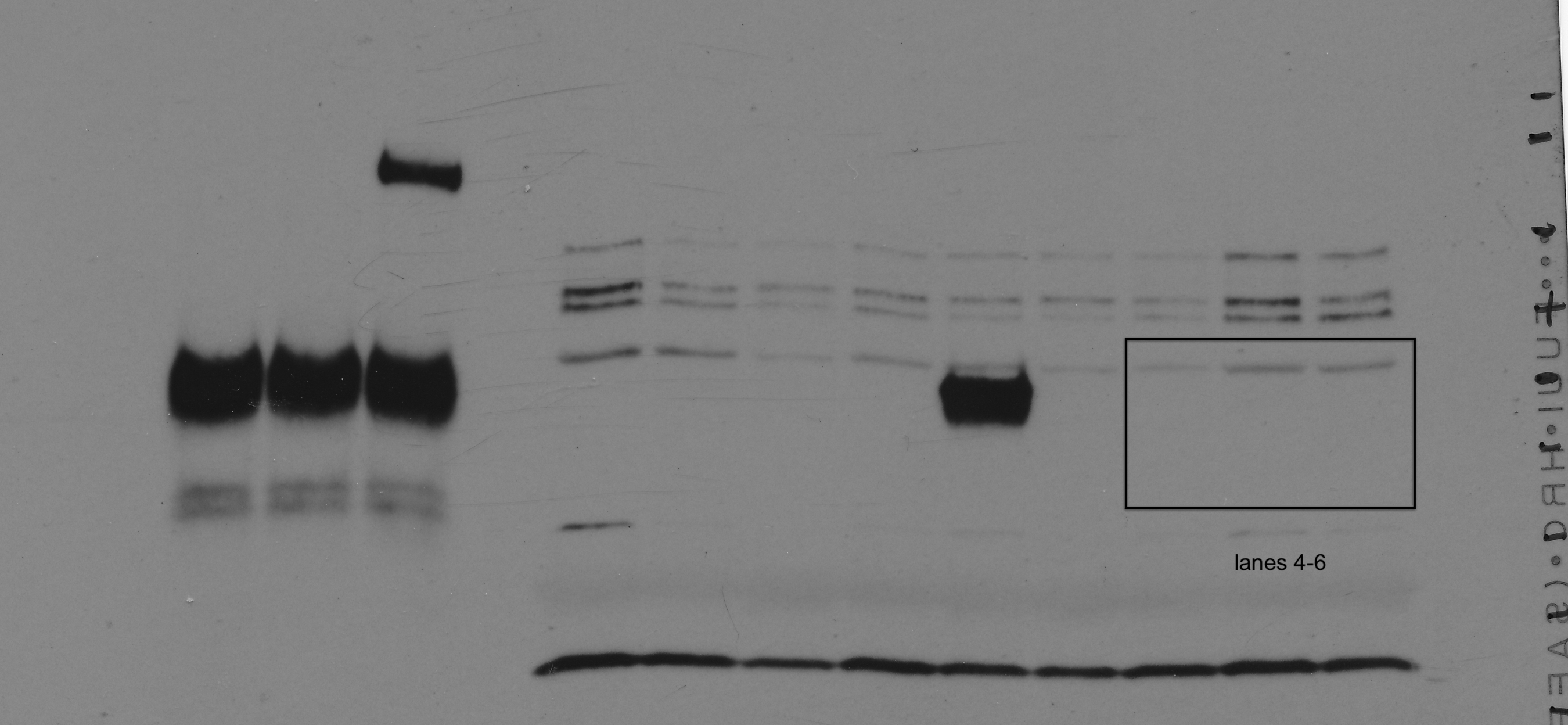

Supplement: Figure 7—figure supplement 1—source data 1. [file elife-83951-fig7-figsupp1-data1.zip › Figure S5 IP CEBPA, IB antiDMA, +PRMT1 marked.tif]

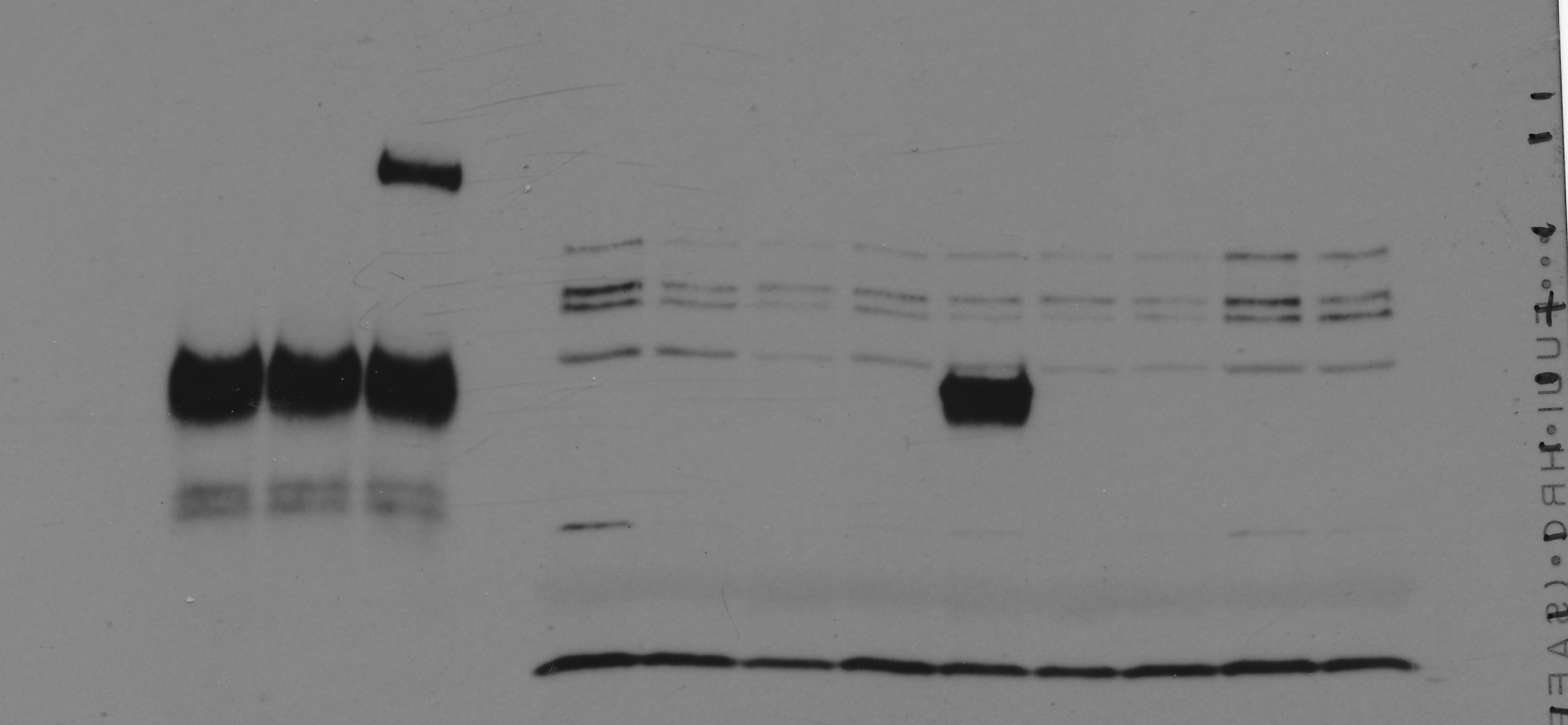

Supplement: Figure 7—figure supplement 1—source data 1. [file elife-83951-fig7-figsupp1-data1.zip › Figure S5 IP CEBPA, IB antiDMA, +PRMT1 source.tif]

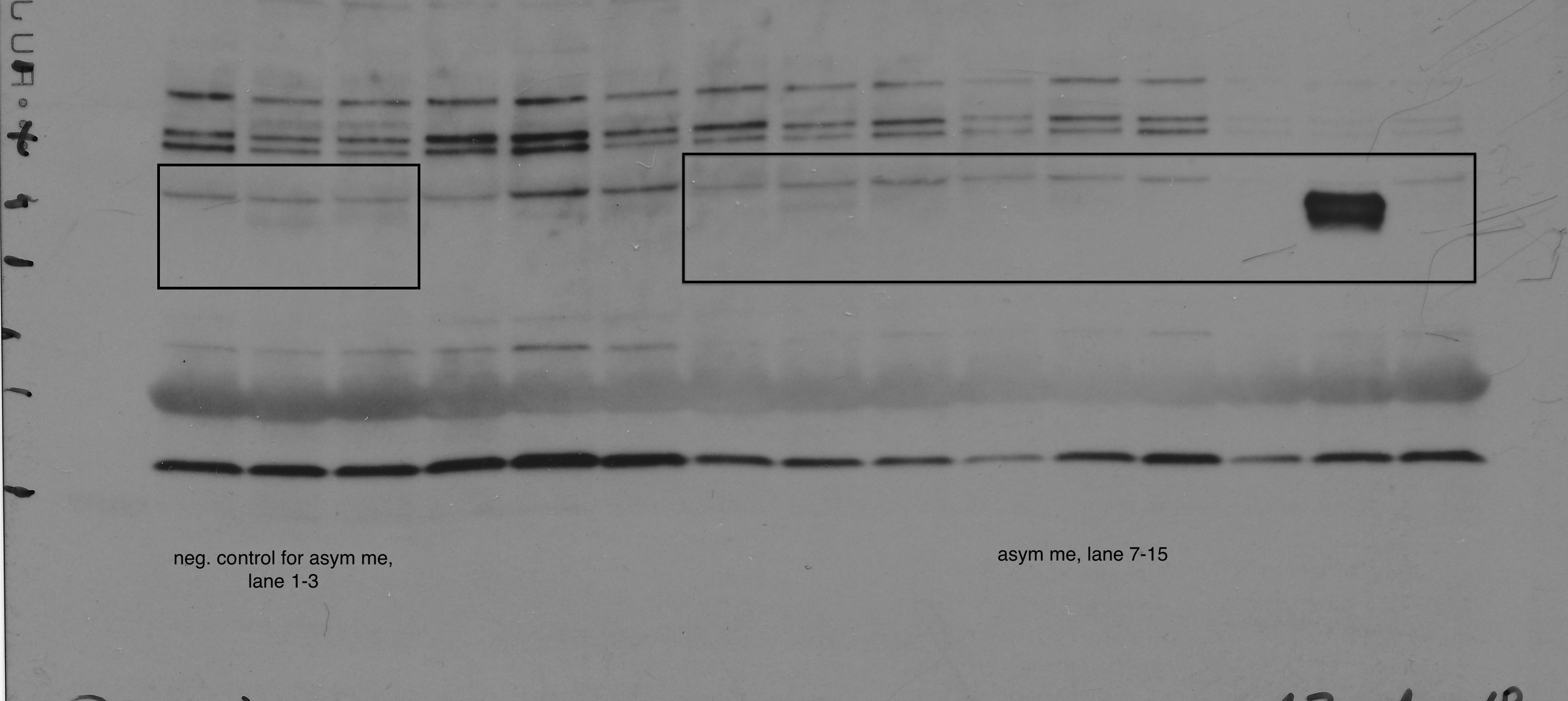

Supplement: Figure 7—figure supplement 1—source data 1. [file elife-83951-fig7-figsupp1-data1.zip › Figure S5 IP CEBPA, IB antiDMA, -:+PRMTs marked.tif]

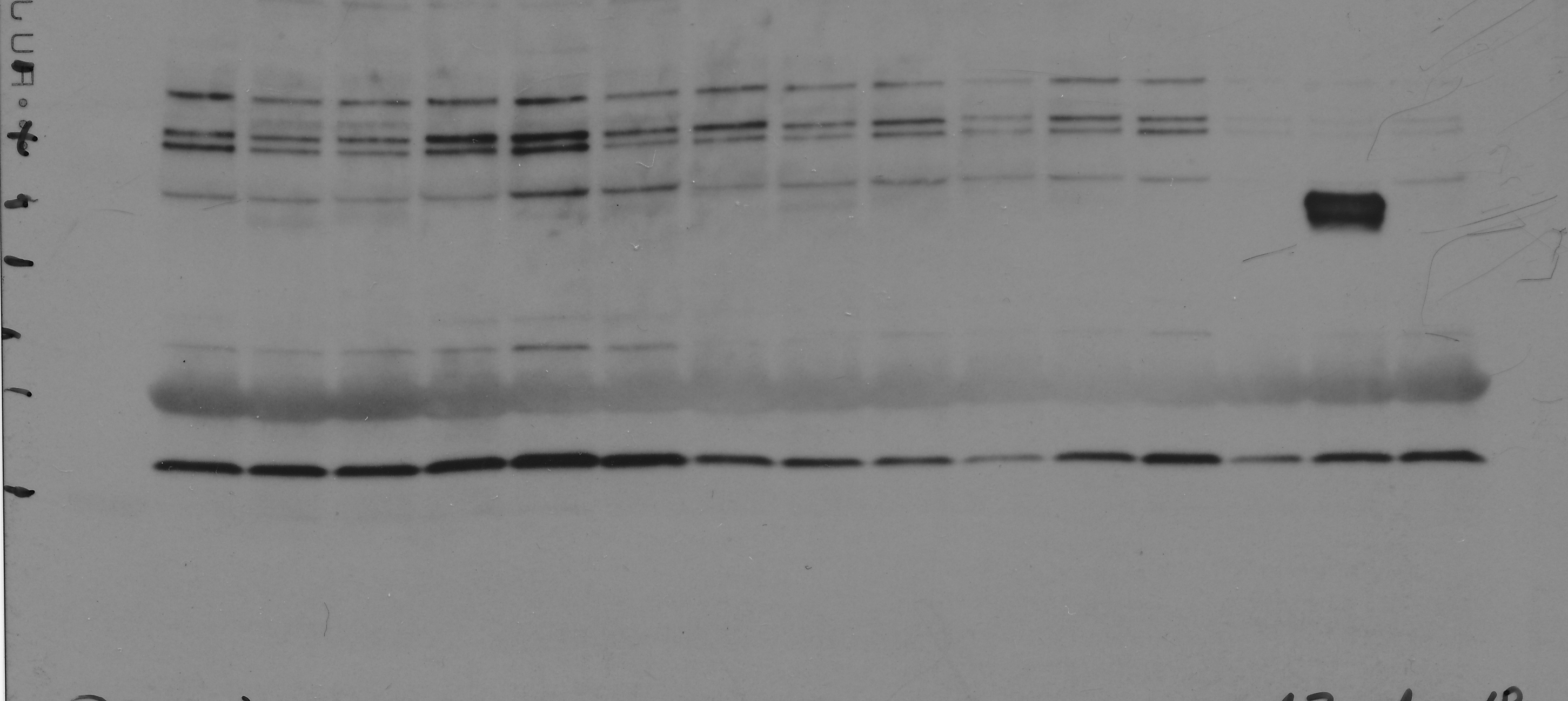

Supplement: Figure 7—figure supplement 1—source data 1. [file elife-83951-fig7-figsupp1-data1.zip › Figure S5 IP CEBPA, IB antiDMA, -:+PRMTs source.tif]

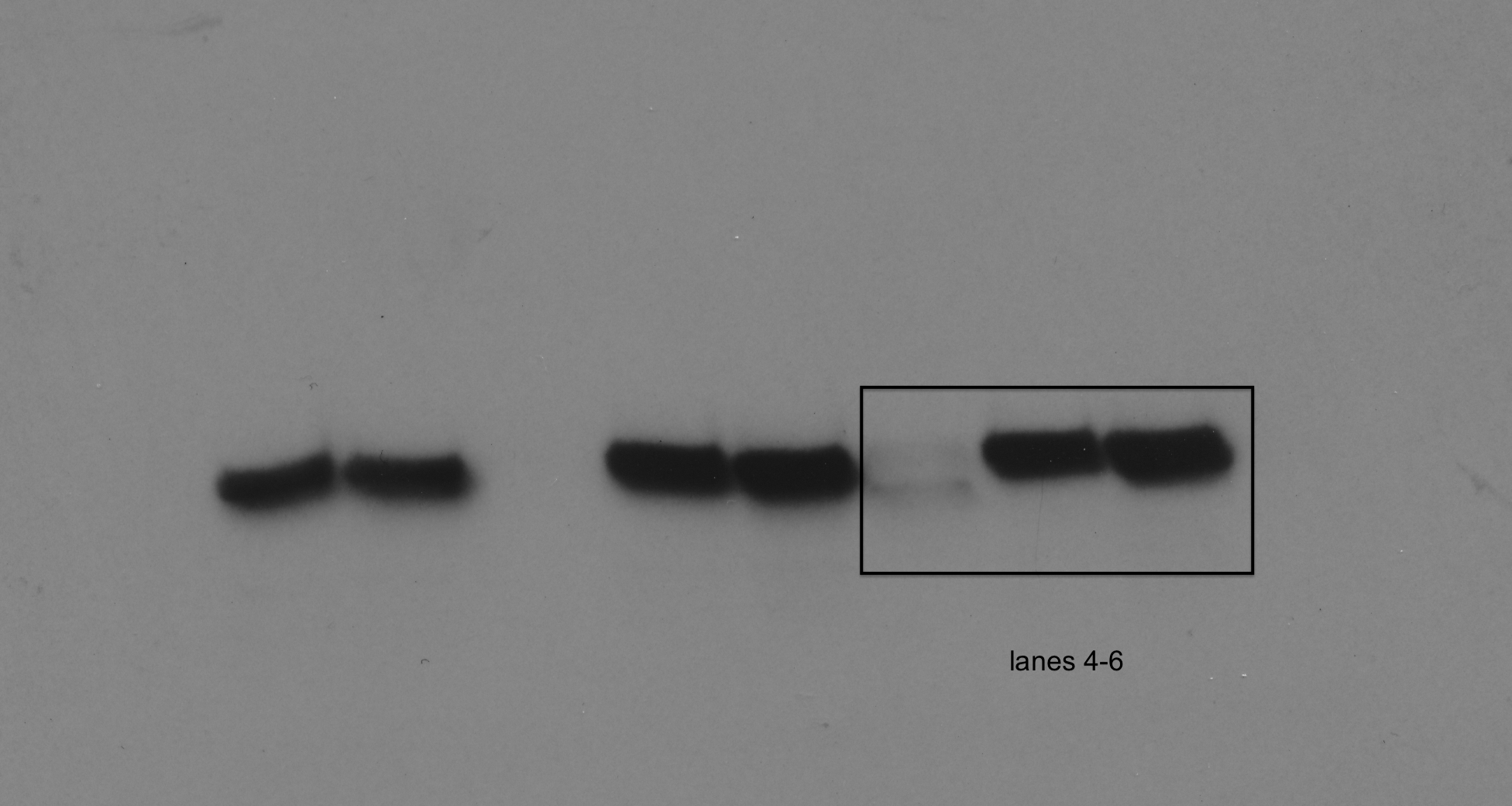

Supplement: Figure 7—figure supplement 1—source data 1. [file elife-83951-fig7-figsupp1-data1.zip › Figure S5 IP CEBPA, IB antiFLAG, +PRMT1 marked.tif]

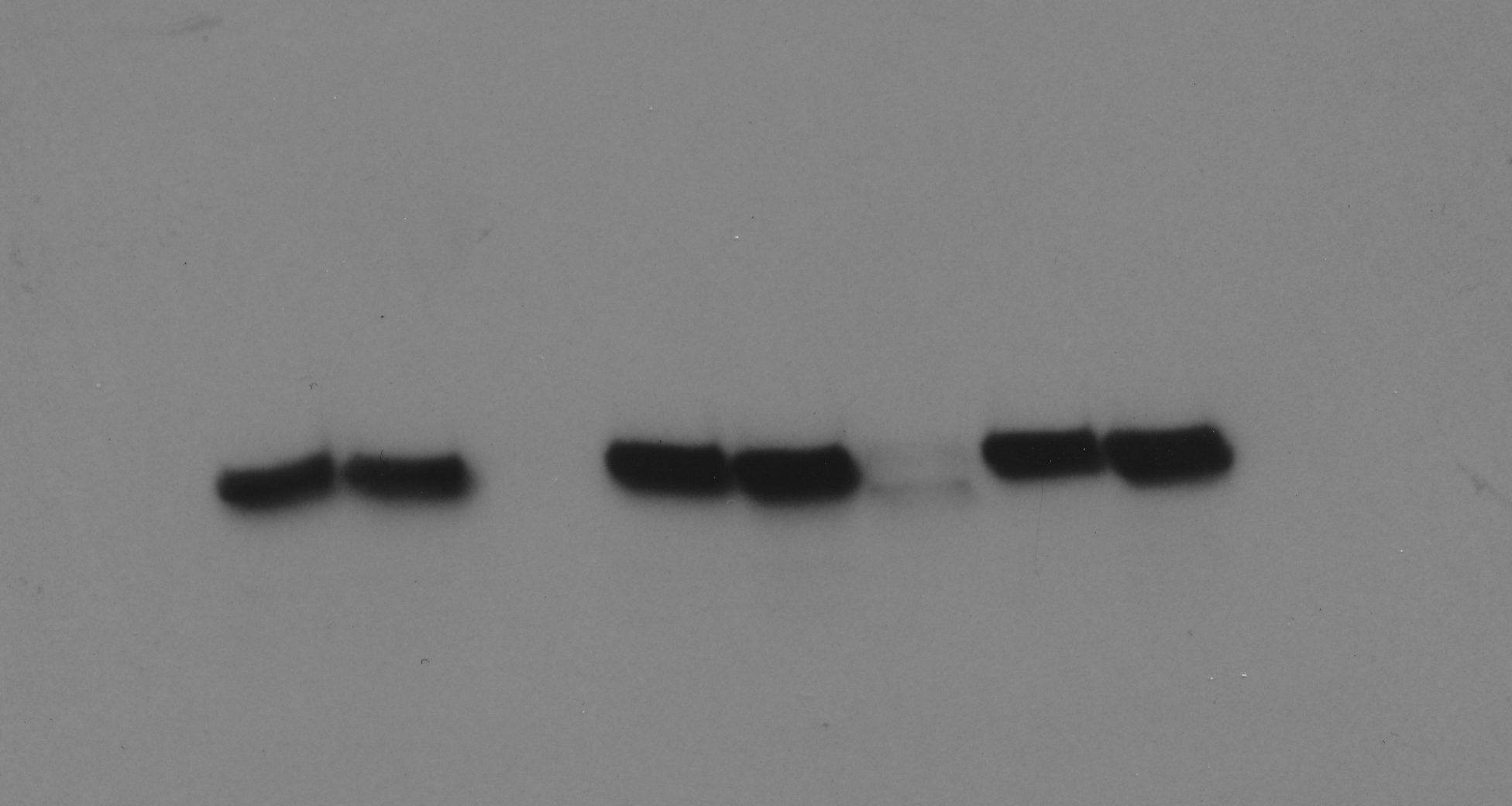

Supplement: Figure 7—figure supplement 1—source data 1. [file elife-83951-fig7-figsupp1-data1.zip › Figure S5 IP CEBPA, IB antiFLAG, +PRMT1 source.tif]

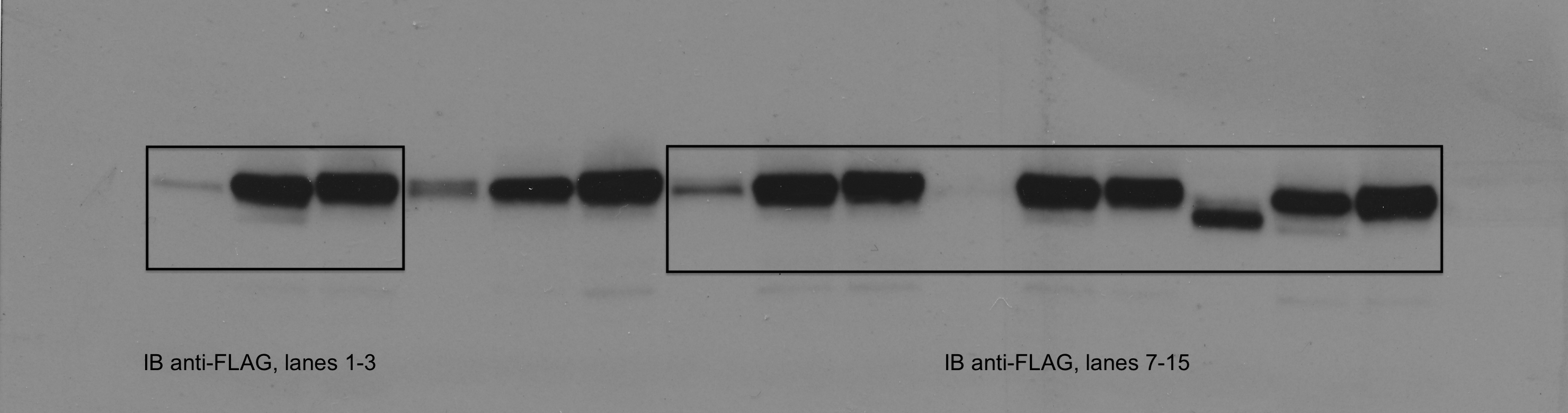

Supplement: Figure 7—figure supplement 1—source data 1. [file elife-83951-fig7-figsupp1-data1.zip › Figure S5 IP CEBPA, IB antiFLAG,-:+PRMTs marked.tif]

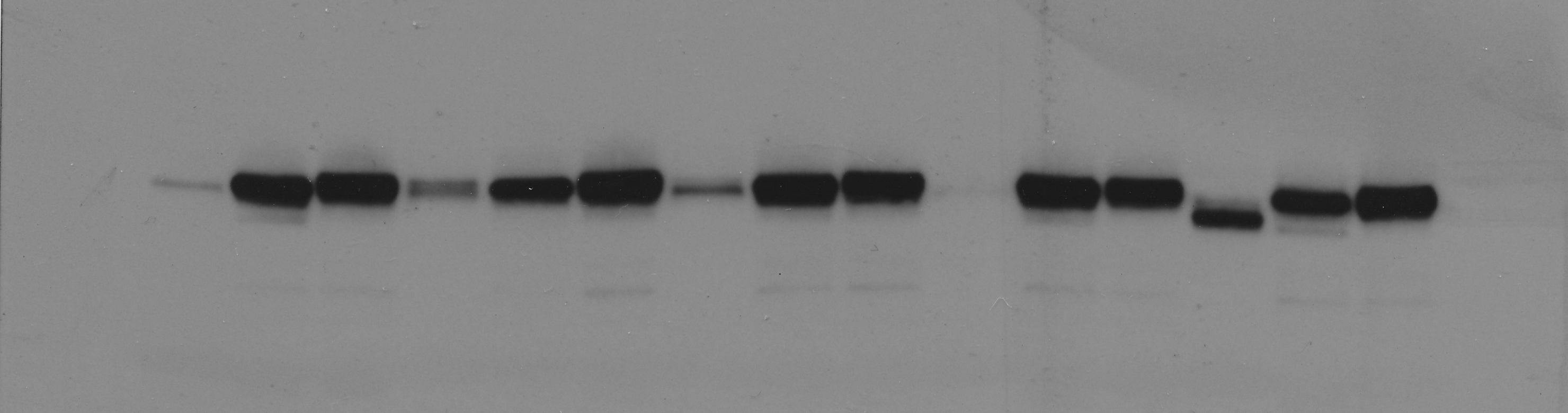

Supplement: Figure 7—figure supplement 1—source data 1. [file elife-83951-fig7-figsupp1-data1.zip › Figure S5 IP CEBPA, IB antiFLAG,-:+PRMTs source.tif]

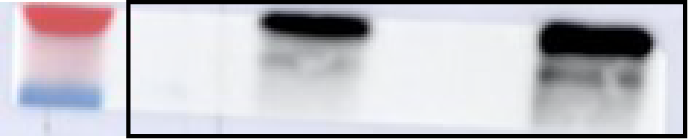

Supplement: Figure 7—figure supplement 1—source data 2. [file elife-83951-fig7-figsupp1-data2.zip › Figure_S5D_source/FigureS5D_Carm1_marked.tif]

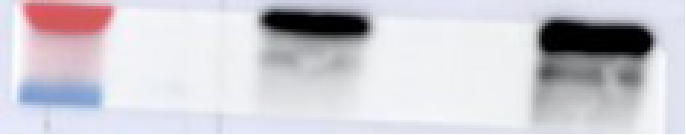

Supplement: Figure 7—figure supplement 1—source data 2. [file elife-83951-fig7-figsupp1-data2.zip › Figure_S5D_source/FigureS5D_Carm1_Source.tif]

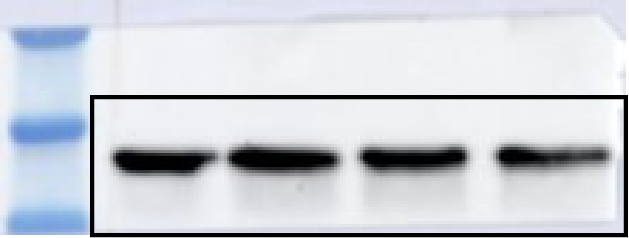

Supplement: Figure 7—figure supplement 1—source data 2. [file elife-83951-fig7-figsupp1-data2.zip › Figure_S5D_source/FigureS5D_GAPDH_marked.tif]

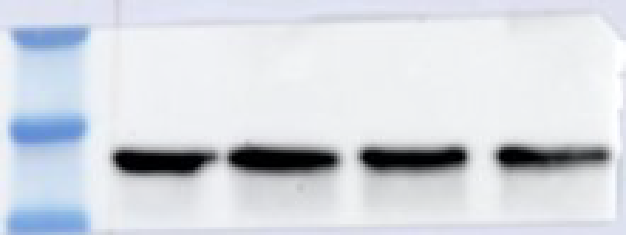

Supplement: Figure 7—figure supplement 1—source data 2. [file elife-83951-fig7-figsupp1-data2.zip › Figure_S5D_source/FigureS5D_GAPDH_Source.tif]

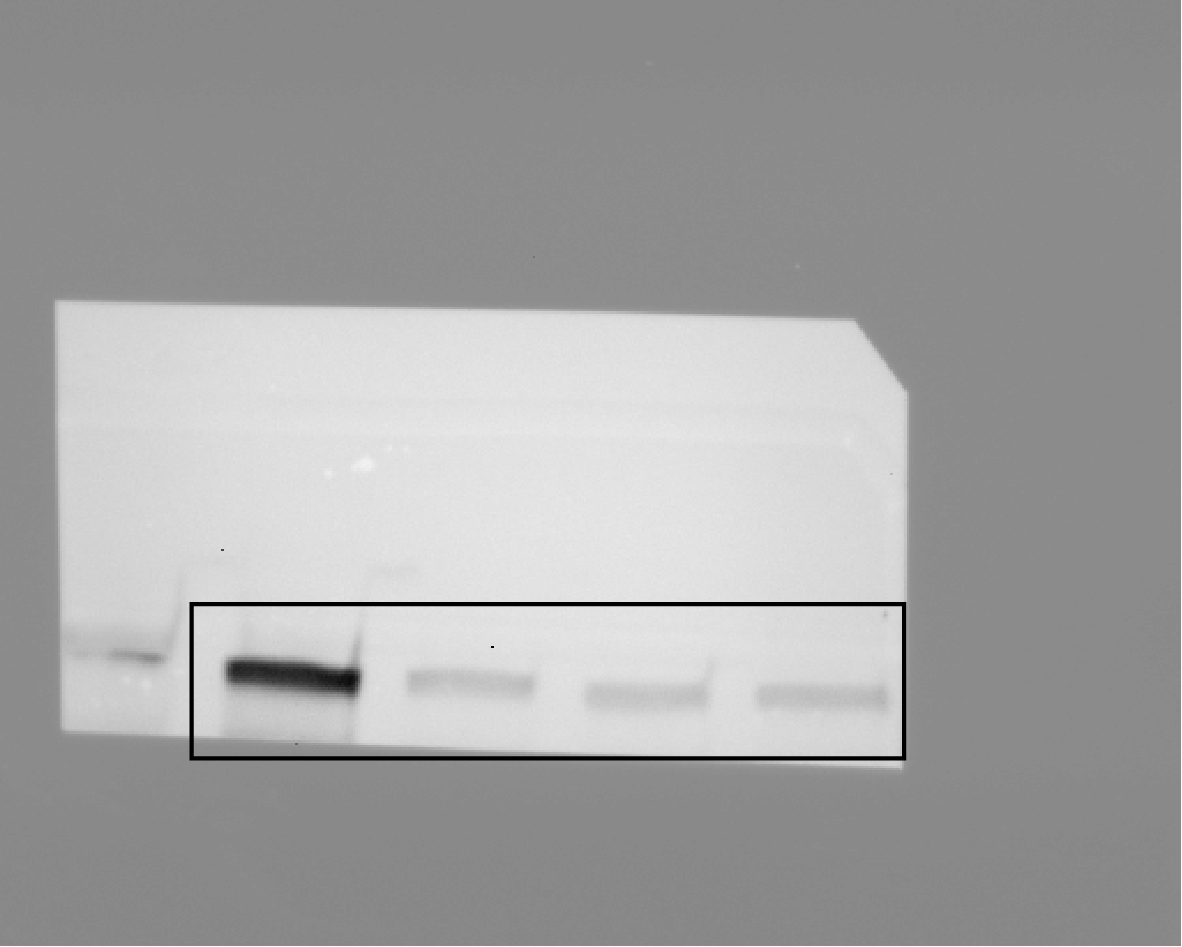

Supplement: Figure 7—figure supplement 1—source data 3. [file elife-83951-fig7-figsupp1-data3.zip › Figure_S5E_source/FigS5E_AsDMBAF155_marked.tif]

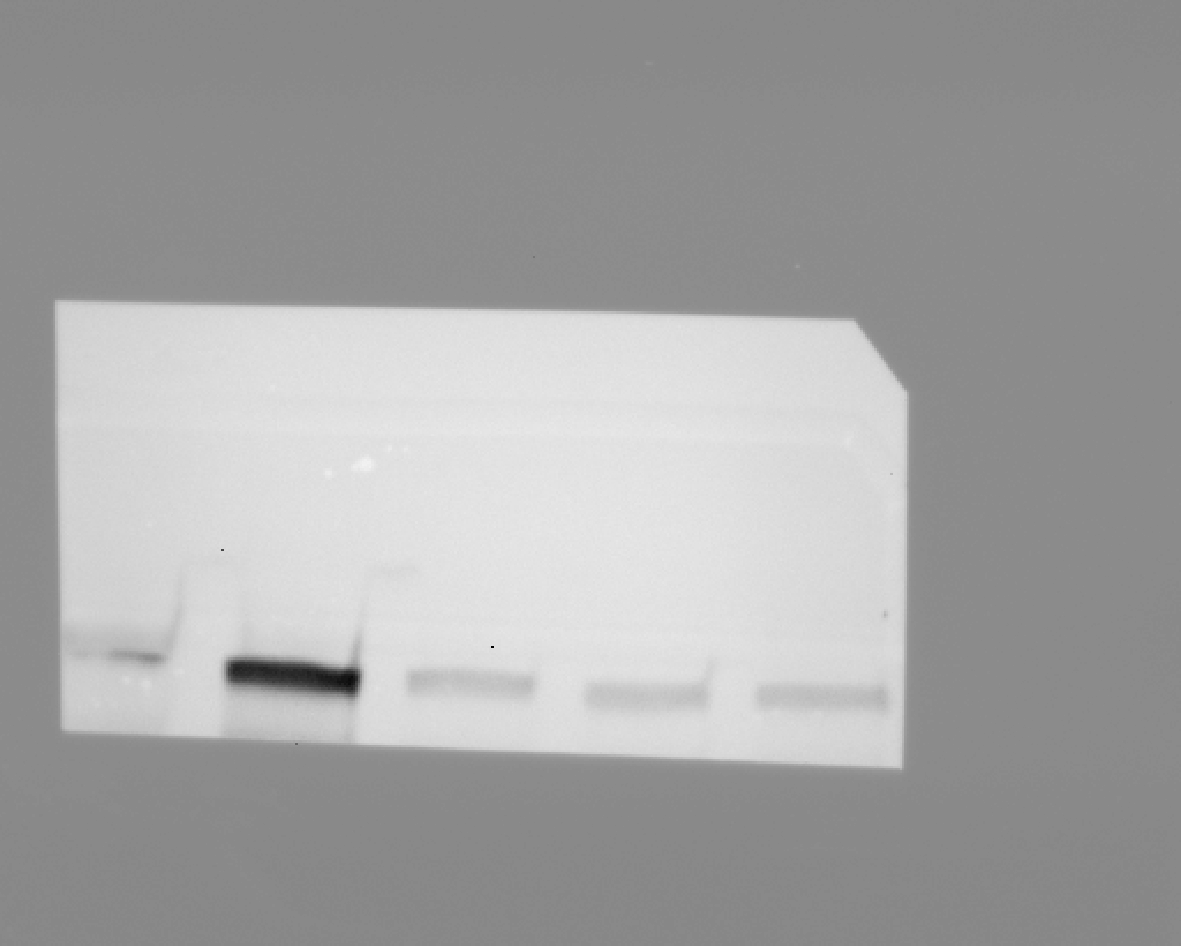

Supplement: Figure 7—figure supplement 1—source data 3. [file elife-83951-fig7-figsupp1-data3.zip › Figure_S5E_source/FigS5E_AsDMBAF155_source.tif]

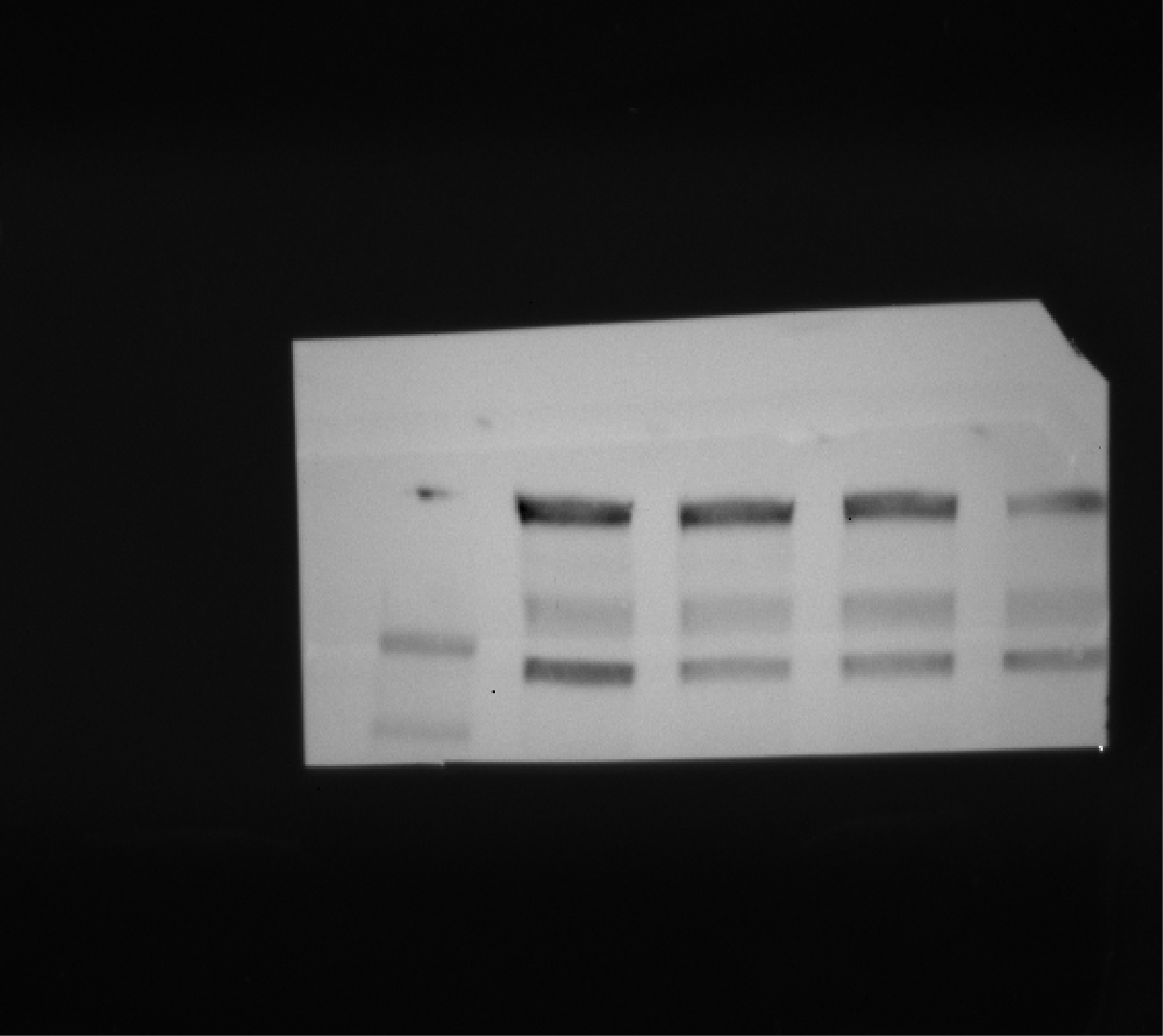

Supplement: Figure 7—figure supplement 1—source data 3. [file elife-83951-fig7-figsupp1-data3.zip › Figure_S5E_source/FigS5E_BAFF155.tif]

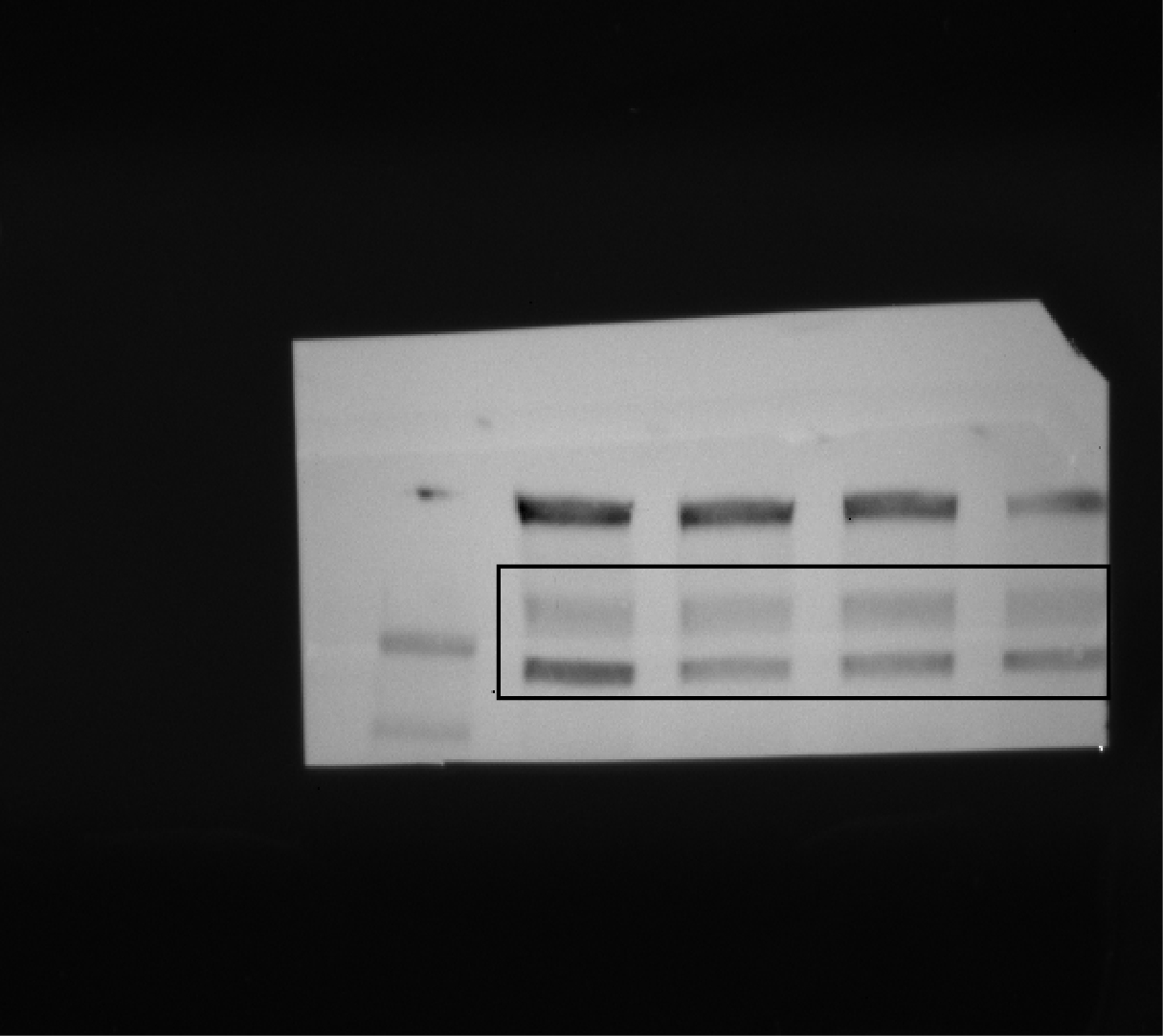

Supplement: Figure 7—figure supplement 1—source data 3. [file elife-83951-fig7-figsupp1-data3.zip › Figure_S5E_source/FigS5E_BAFF155_marked.tif]

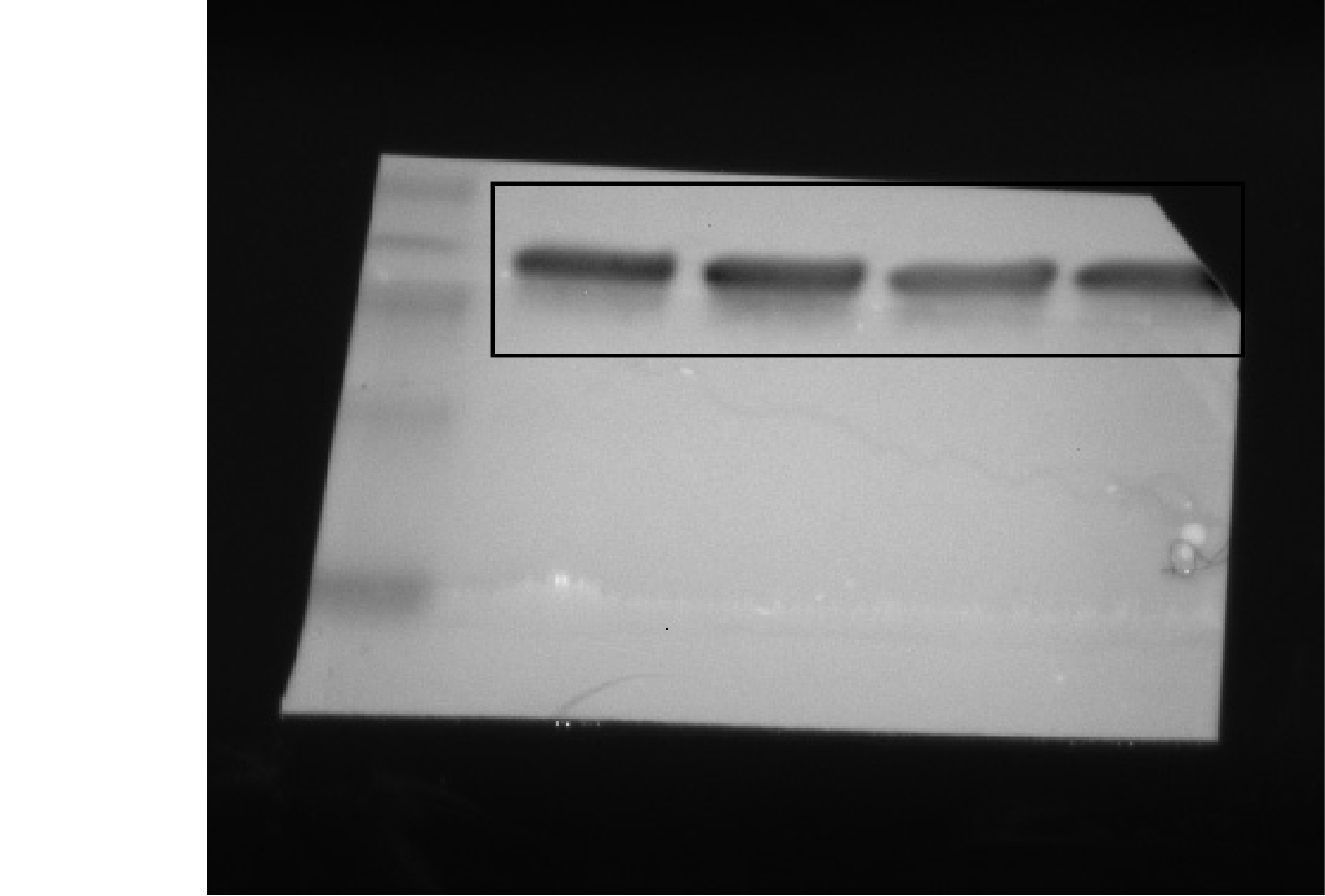

Supplement: Figure 7—figure supplement 1—source data 3. [file elife-83951-fig7-figsupp1-data3.zip › Figure_S5E_source/FigS5E_GAPDH_marked.tif]

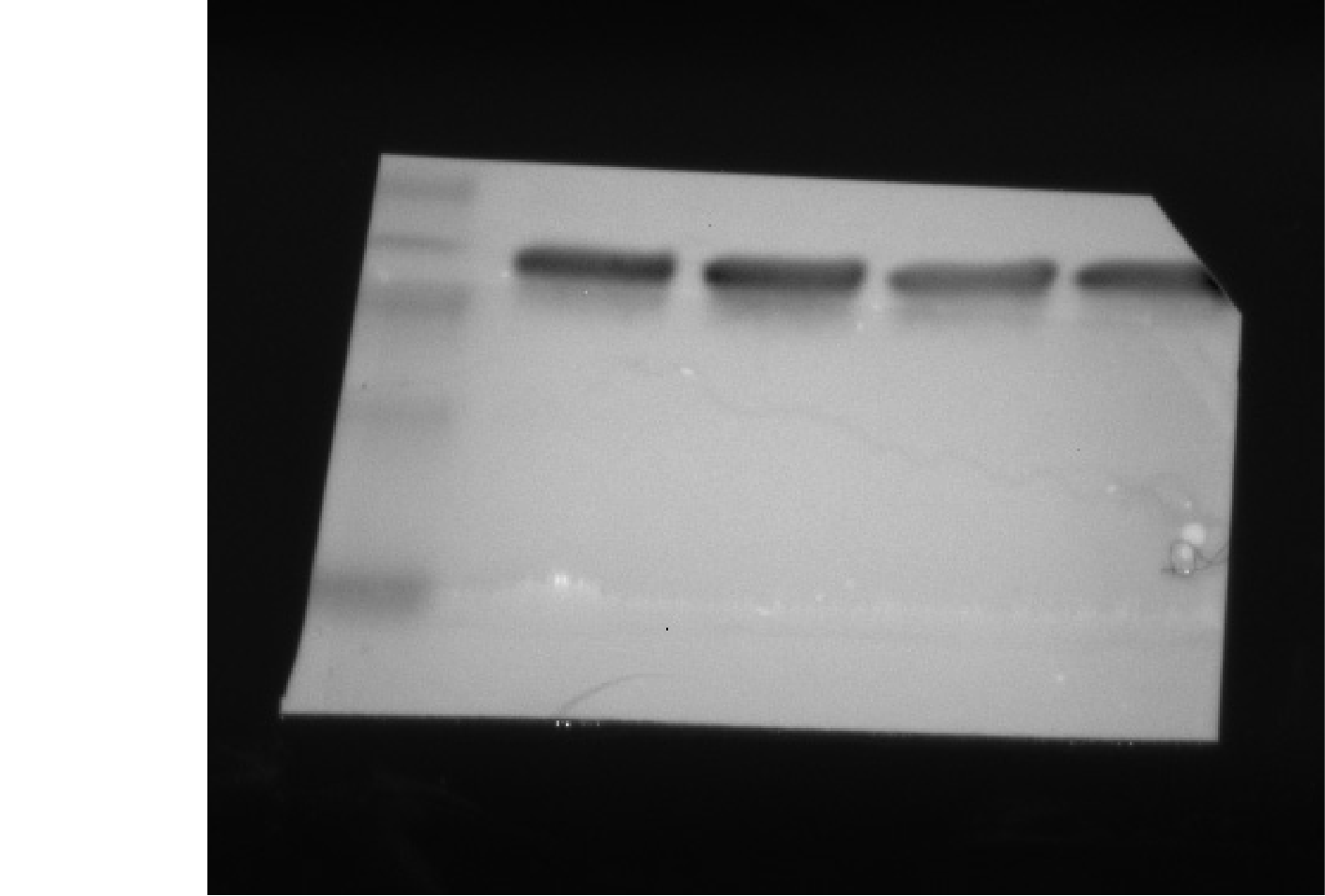

Supplement: Figure 7—figure supplement 1—source data 3. [file elife-83951-fig7-figsupp1-data3.zip › Figure_S5E_source/FigS5E_GAPDH_source.tif]
