# Supplementary material for: Carm1-arginine methylation of the transcription factor C/EBPα regulates transdifferentiation velocity
Source: eLife. 2023 Jun 27;12:e83951. doi: 10.7554/eLife.83951 (PMC10299824; doi:10.7554/eLife.83951)
Supplement: Supplementary file 1. — The table lists the antibodies used for the experiments and the sources. Related to Figure 1A, C and D; Figure 2A; Figure 1—figure supplement 1A and Figure 7—figure supplement 2G,H [file elife-83951-supp1.docx]

**Supplementary file 1**

**Antibodies used for cell sorting and Flow cytometry experiments**

|  | | | | |
| --- | --- | --- | --- | --- |
| **Antibody** | **Company** | **Catalogue** | **Species** | **Dilution** |
| CD16/CD32  (FcBlock) | BD Pharmingen | 553142 | Rat | 1:400 |
| CD19-Biotin | BD Biosciences | 553784 | Rat | 1:400 |
| Mac1-Biotin | BD Pharmingen | 557395 | Rat | 1:400 |
| hCD4-Biotin | eBioscience | 13-0049 | Mouse | 1:33 |
| CD19-APC | BD Pharmingen | 550992 | Rat | 1:400 |
| Mac1-PE-Cy7 | BD Pharmingen | 552850 | Rat | 1:400 |
| Ly6g-PE | Pharmingen | 553128 | Rat | 1:400 |
| Mac1-APC | eBioscience | 17-0112-83 | Rat | 1:400 |
| hCD4-PE | BD Pharmingen | 555347 | Mouse | 1:20 |
| hCD16/CD32  (hFcBlock) | Invitrogen | 16-9161-73 | - | 1:20 |
| hCD19-APC-Cy7 | BD Pharmingen | 557791 | Mouse | 1:33 |
| hMac1-APC | BD Pharmingen | 561015 | Mouse | 1:33 |
| CD16/CD32-FITC | BD Pharmingen | 553144 | Rat | 1:400 |
| cKit-APC-Cy7 | Invitrogen | 47-1172-82 | Rat | 1:400 |
| CD34-APC | BD Pharmingen | 560230 | Rat | 1:50 |
| Sca1-PE-Cy7 | BD Pharmingen | 558162 | Rat | 1:400 |
| Sca1-PerCP-Cy5.5 | eBioscience | 35-5981-82 | Rat | 1:400 |
| CD41-PE-Cy7 | eBioscience | 25-0411-82 | Rat | 1:400 |

| **Intracellular staining for flow cytometry** | | | | |
| --- | --- | --- | --- | --- |
| **Antibody** | **Company** | **Catalogue** | **Species** | **Dilution** |
| C/EBPα | Cell Signaling | 8178 | Rabbit | 1:100 |
| Carm1 | Cell Signaling | 12495 | Mouse | 1:100 |
| PU.1 | Abcam | Ab88082 | Mouse | 1:100 |
| BAFF155 | Cell Signaling | D7F8S | Rabbit | 1:200 |
| BAFF155-AsDM | Cell Signaling | 94962 | Rabbit | 1:200 |
| AF488 Anti-rabbit | ThermoFisher | A-11070 | Goat | 1:500 |
| AF555 Anti-mouse | ThermoFisher | A-21422 | Goat | 1:500 |
