# Supplementary material for: Carm1-arginine methylation of the transcription factor C/EBPα regulates transdifferentiation velocity
Source: eLife. 2023 Jun 27;12:e83951. doi: 10.7554/eLife.83951 (PMC10299824; doi:10.7554/eLife.83951)
Supplement: Supplementary file 2. — The table lists the chemical reagents used for western blot experiments. (Figures 3C;; 7A, B, C; Figure 4—figure supplement 1C; Figure 7—figure supplement 2A,D,E). [file elife-83951-supp2.docx]

**Supplementary file 2**

**Chemical reagents used to prepare buffers for western blot.**

| **Running buffer** | **Transfer buffer** | **TBST** |
| --- | --- | --- |
| 25mM Tris-base | 25mM Tris-HCl pH=3.8 | 10mM Tris HCl=7.5 |
| 200mM glycine | 200mM glycine | 100mM NaCl |
| 0.1% SDS | 20% methanol | 0.1% Tween 20 |
