# Supplementary material for: Carm1-arginine methylation of the transcription factor C/EBPα regulates transdifferentiation velocity
Source: eLife. 2023 Jun 27;12:e83951. doi: 10.7554/eLife.83951 (PMC10299824; doi:10.7554/eLife.83951)
Supplement: Supplementary file 3. — The table lists the antibodies used for western blot experiments and their sources (Figures 3C;; 7A, B, C; Figure 4—figure supplement 1C; Figure 7—figure supplement 2A, D, E). [file elife-83951-supp3.docx]

**Supplementary file 3**

**Antibodies used for western blot experiments**

| **Antibody** | **Company** | **Catalogue** | **Species** | **Dilution** |
| --- | --- | --- | --- | --- |
| C/EBPα | Cell Signaling | 8178 | Rabbit | 1:1000 |
| aDMA | Cell Signaling | 13522S | Rabbit | 1:1000 |
| aDMA | Upstate | #07-414 | Rabbit | 1:1000 |
| HA | Covance | #MMS-101R | Mouse | 1:1000 |
| Flag | Sigma | F3165 | Mouse | 1:1000 |
| Flag | Abnova | PAB 29056 | Chicken | 1:1000 |
| BAFF155 | Cell Signaling | D7F8S | Rabbit | 1:1000 |
| BAFF155-AsDM | Cell Signaling | 94962 | Rabbit | 1:1000 |
| PU.1 | Abcam | Ab88082 | Mouse | 1:1000 |
| Vinculin | Merck | V9131 | Mouse | 1:200 |
| Gapdh | Abcam | Ab8245 | Mouse | 1:5000 |
| H3 | Abcam | Ab10799 | Mouse | 1:1000 |
