# Supplementary material for: Carm1-arginine methylation of the transcription factor C/EBPα regulates transdifferentiation velocity
Source: eLife. 2023 Jun 27;12:e83951. doi: 10.7554/eLife.83951 (PMC10299824; doi:10.7554/eLife.83951)
Supplement: Supplementary file 4. — The table lists the peptides used for the in vitro Carm1 methylation experiments (Figure 7—figure supplement 2C). [file elife-83951-supp4.docx]

**Supplementary file 4**

**Peptides used for in vitro methylation experiments**

| **Peptides** |
| --- |
| YEAEPRPPMSS, aa 7-17 |
| AFGFPRGAGPA, aa 30-40 |
| LFQHSRQQEKA, aa 81-91 |
| GYLDGRLEPLY, aa 137-147 |
| EPLYERVGAPA, aa 144-154 |
| GAPALRPLVIK, aa 151-161 |
| IKQEPREEDEA, aa 160-170 |
| AHPDLRASGGS, aa 259-269 |
| SNEYRVRRERNNIA, aa 282-295 |
| NIAVRKSRDKAK, aa 293-304 |
| DKAKQRNVETQ, aa 301-311 |
| SDNDRLRKRVEQL, aa 319-331 |
| VEQLSRELDTL, aa 328-338 |
| ELDTLRGIFRQLPES, aa 334-348 |
| MSSHLQSPPHAPSSAAFGFPRGAGPAQPPAPPAAPEPLGG aa 15-54 |
| MSSHLQSPPHAPSSAAFGFPR(me2)GAGPAQPPAPPAAPEPLGG aa 15-54 |
| MSSHLQSPPHAPSSAAFGFPR/AGAGPAQPPAPPAAPEPLGG aa 15-54 |
| PRMT4 peptide substrate |
| Histone H3 aa 1-21 |
| Histone H4 aa 1-21 |
